# Livestock and food security: vulnerability to population growth and climate change

**DOI:** 10.1111/gcb.12589

**Published:** 2014-05-02

**Authors:** Olivia F Godber, Richard Wall

**Affiliations:** Veterinary Parasitology and Ecology Group, University of BristolBristol Life Sciences Building, Bristol, BS8 1TQ, UK

**Keywords:** climate change, food security, livestock, population growth, vulnerability

## Abstract

Livestock production is an important contributor to sustainable food security for many nations, particularly in low-income areas and marginal habitats that are unsuitable for crop production. Animal products account for approximately one-third of global human protein consumption. Here, a range of indicators, derived from FAOSTAT and World Bank statistics, are used to model the relative vulnerability of nations at the global scale to predicted climate and population changes, which are likely to impact on their use of grazing livestock for food. Vulnerability analysis has been widely used in global change science to predict impacts on food security and famine. It is a tool that is useful to inform policy decision making and direct the targeting of interventions. The model developed shows that nations within sub-Saharan Africa, particularly in the Sahel region, and some Asian nations are likely to be the most vulnerable. Livestock-based food security is already compromised in many areas on these continents and suffers constraints from current climate in addition to the lack of economic and technical support allowing mitigation of predicted climate change impacts. Governance is shown to be a highly influential factor and, paradoxically, it is suggested that current self-sufficiency may increase future potential vulnerability because trade networks are poorly developed. This may be relieved through freer trade of food products, which is also associated with improved governance. Policy decisions, support and interventions will need to be targeted at the most vulnerable nations, but given the strong influence of governance, to be effective, any implementation will require considerable care in the management of underlying structural reform.

## Introduction

By the end of 2011, the global population exceeded 7 billion and it is expected to reach between 8.1 billion and 10.6 billion by 2050 (UN, 2011). In addition to this growth in the number of people requiring food, rising incomes and increasing food demand will result in the need for important changes to ensure food security (UN, 2011). With 12.5% of the world's population undernourished with respect to energy intake (FAO, WFP, IFAD, 2012), it is predicted that to meet the food demands of a population of 9 billion people, food production will need to increase by 70% (UN, 2011). Livestock production will inevitably play a contributory role in achieving this sustainable food security, influenced strongly by cultural predilections (Devendra, 2001); meat demand per capita is expected to rise by almost 13% in developing nations over the period 2008–2017 (OECD-FAO, 2008). Livestock is considered a key asset in low-income areas, acting as a crucial food resource in the case of crop failures (IFAD, 2007) with ruminant-based products increasing the human food supply of highly protein-rich products (Kabubo-Mariara, 2009). Animal products in general account for approximately one-third of global human protein consumption (Steinfeld *et al.*, 2006; Popp *et al.*, 2010). Livestock husbandry on grassland systems also give benefits which include the conservation of rangeland ecosystems, promotion of the use of land-preserving forages and the production of food from land that would be unsuitable for crop production (Janzen, 2011).

The sustainability of livestock husbandry, however, is affected by a wide range of environmental challenges, not the least of which is climate change (Kabubo-Mariara, 2009; Wall & Ellse, 2011). A clear, strategic and long-term understanding of the challenges of climate change is needed, on a global scale, to allow its appropriate management (Gjerris *et al.*, 2011). The main consequences resulting from climate change are likely to include altered rangeland productivity, effects on livestock parasites and disease and increased competition for both land and water; location, production system, crop and pasture species will determine the extent and direction of the anticipated effects (Thornton & Gerber, 2010). Effects of climate change on the prevalence of parasites and infectious disease are likely to be particularly important, mediated through altered development rates of pathogens and parasites, shifts in disease distribution affecting susceptible or naive animal populations, changes to the distribution and abundance of disease vectors and epidemiological effects, such as altered transmission rates between hosts (Baylis & Githeko, 2006; Wall *et al.*, 2011). Along with anticipated changes in climate, intensification of production systems and the extension and growing complexity of market chains to meet product demand will also affect disease risks (Randolph, 2008). However, predictions of likely changes in livestock disease are relatively crude, due largely to a lack of knowledge of the relationship between livestock disease and environmental factors under current conditions, let alone those which may apply in the future (Thornton & Gerber, 2010).

One approach that has been widely used in global change science and in studies of food security and famine is vulnerability analysis (Watts & Bohle, 1993; Smit & Wandel, 2006). It is a tool that can be used to predict likely impacts on coupled human–environment systems and can be used to inform and guide policy decision making and help target interventions (Smit & Wandel, 2006). Although related, measures of vulnerability are different to estimates of criticality; a system can exhibit criticality without the population necessarily being vulnerable as a result of its resilience and sensitivity to the shock or hazard (Adger, 2000; Turner *et al.*, 2003). Vulnerability assessment allows the degree to which a system is likely to be degraded by environmental challenge to be assessed and is considered to be more informative than the use of risk-hazard models (Turner *et al.*, 2003). Vulnerability is usually considered to be the product of three elements: sensitivity, exposure and adaptive capacity (Smit & Wandel, 2006). These elements are interactive and scale dependent and should ideally capture the essence of the system of interest (Turner *et al.*, 2003), although simplification is usually inevitable. Sensitivity is the intrinsic degree to which biophysical, social and economic factors are likely to be influenced by extrinsic stresses or hazards, but can also represent the dependence on a specific driver and its importance to a sector, for instance the economy (Allison *et al.*, 2009). Measures of exposure attempt to capture the extent to which a system will be influenced by any specific change. Adaptive capacity aims to capture the ability of a system to undertake mitigating responses. Adaptations can take several forms, including technological, behavioural, managerial and policy based (Thornton *et al.*, 2009) and hence, this index typically includes information on the financial strength of the government and industry, communications infrastructure and per capita affluence (Haddad, 2005).

The aim of the work described here was to use a range of measures relevant to socioeconomic status, food security and livestock production systems, for as wide a range of nations as possible, to estimate the sensitivity, exposure and adaptive capacity of their use of grazing livestock to the anticipated effects of climate change and population growth. These three indices would then be used to estimate the potential vulnerability of a nation and their livestock sector to climate and population change. For this study, sensitivity is defined as a nation's nutritional reliance on grazing animal-based food products and level of food security; exposure is defined as the projected changes in climate and population growth; adaptive capacity is a measure of a nation's ability to change in response to or cope with changes in climate and food demand.

## Materials and methods

### Selection of indicators

FAOSTAT and World Bank data banks were searched for suitable quantitative indicators relevant to grazing animal production, food security, climate change, population growth and socio-economic status for the years 2009, 2010 or 2011. For the purposes of this study, grazing animals included cattle, sheep, goats, buffalo, camels and other camelids, horses, donkeys and asses; essentially ruminants and equids dependent on forage and pastureland. Nations not appearing in both data banks were excluded. For the 2009–2011 period, the FAO and World Bank recognized 231 and 214 nations respectively. Of these, 208 appear in both data banks. Where possible, data from 2010 were used; if unavailable, 2009 or 2011 data were substituted to maximize the number of indicators that could be used and nations available for inclusion in the analysis.

Sufficient data were considered to be available for an initial 76 informative indicators. Indicators based on livestock numbers were converted to livestock units, based on Chilonda & Otte (2006), to allow for differences between livestock species (where a cow in the United States has a value of 1 and a sheep a value of 0.15, for example). For the indicators included in the estimate of exposure to climate change, climate change projections for 2045–2065 were taken from an ensemble of data from nine general circulation models run under the IPCC A2 scenario (World Bank, 2012). Annual averages are given relative to the period 1961–2000 and aggregated to country level from 2-degree gridded data for precipitation and temperature variables (World Bank, 2012). Changes in precipitation and temperature will have extremely varied impacts on animal and forage production in different nations, with both positive and negative effects being seen depending on their individual circumstances. For instance, a reduction in the level of precipitation may help reduce the incidence of some animal diseases, but simultaneously may reduce grassland productivity (IFAD, 2007). Consequently, the exposure scores focus on the degree of change predicted for a nation; the greater the change in a climatic variable, whether an increase or a decrease, the higher the score. A similar compromise was made in the monotonic model of Rees *et al*. (2008). Each indicator was scaled from zero to one on a linear, absolute scale, to allow for the different units in which the indicators were recorded, where zero was the lowest value seen and one the highest. Indicators were not normalized so that the true distribution in indicator values would be reflected in the final vulnerability scores.

Initially, preliminary models were constructed using all 76 indicators. Then, to produce minimal models, all the indicators were allocated to a number of meaningfully related subject categories. First, for the climate change indicators, the absolute projected change for temperature (Δ*t*) was multiplied by the absolute projected change for precipitation (Δ*p*) and weighted through further multiplication by the percentage of a nation's population affected by droughts, flooding and extreme weather events in the preceding 20 years (*w*). This gave a single value (*cc*) to estimate the likely impact of climate on livestock production: (1)
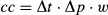


Within each of the remaining subject categories, all indicators were correlated with each other using Spearman rank correlation. The single indicator that correlated most strongly with others within the same category was retained (see Table1). This resulted in a single indicator for each of eight categories across a total of 148 nations (Table1), which were used to build the final minimal models.

**Table 1:** Summary of indicators used to calculate sensitivity, exposure and adaptive capacity and the source of the data

Index	Indicator		Source
Sensitivity – nutritional reliance on home-produced grazing animal-based food products and level of food security. Grazing animals include cattle, sheep, goats, buffaloes, camels and other camelids, horses, donkeys and asses; essentially ruminants and equids dependent on forage and pasture land	Self-sufficiency	Consumption of home-produced grazing animal-based food products as a proportion of all consumed animal-based food products: [(production − exports)/(production − exports + imports)]	FAOSTAT (2013)
	Nutritional contribution	Contribution of grazing animal-based food products to nutritional intake from all food products. Captures potential differences between nations according to their diet which may be influenced by the availability and accessibility of different food products, in addition to social factors such as cultural and religious beliefs	FAOSTAT (2013)
	Food insecurity	Prevalence of food inadequacy. A reflection of food availability, stability, utilization and access	FAOSTAT (2013)
Exposure – projected levels for climate change and population growth. Climate change values taken as absolute values to indicate the degree of predicted change	Precipitation	Projected change in annual average precipitation (2045–2065)	World Bank (2012)
	Temperature	Projected change in annual average temperature (2045–2065)	World Bank (2012)
	Extreme weather	Population affected by droughts, flooding and extreme weather (1990–2009)	World Bank (2012)
	Population growth	Projected population change (2010–2050)	FAOSTAT (2013)
Adaptive capacity – nations' abilities to change in response to or cope with changes in climate and food demand	Health	Life expectancy	World Bank (2013)
	Economy	Total GDP	World Bank (2013)
	Governance	Control of corruption	World Bank (2013)
		Government effectiveness	World Bank (2013)
		Political stability and absence of violence/terrorism	World Bank (2013)
		Regulatory quality	World Bank (2013)
		Rule of law	World Bank (2013)
		Voice and accountability	World Bank (2013)

### Modelling vulnerability

In the minimal models, Sensitivity (*S*) was calculated as the sum of self-sufficiency indicator score (*ss*), food security indicator score (*fs*) and nutritional contribution of grazing livestock products to diet indicator score (*nc*), divided by three to give an average score. Exposure (*E*) was calculated as the sum of the climate change indicator score (*cc*) and population growth indicator score (*pg*), divided by two to give an average score. Finally, Adaptive Capacity (*AC*) was calculated as the sum of health (*he*), economy (*ec*) and governance (*gv*) indicator scores, divided by three to give an average score. These were then rescaled from zero to one as before. To obtain an index of vulnerability, both additive [Eqn (2)] and multiplicative [Eqn (3)] minimal models were constructed: (2)
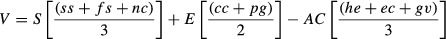

(3)
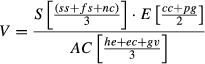


In the additive model presented here, the sensitivity, exposure and adaptive capacity indices contribute equally to the final vulnerability score [Eqn (2)], which can have the disadvantage that an extreme value in one index is offset by counterbalancing values in one or both of the other indices. In contrast, in the multiplicative model [Eqn (3)], extreme values will be reflected disproportionately in the final estimate of vulnerability (Hajkowicz, 2006), which may be important in highlighting scenarios where large changes in any one index have a major impact on the system as a whole.

In each minimal model, vulnerability was again expressed on a scale from zero to one to allow comparison of nations; a score of zero represents a very low and one a very highly vulnerable nation. For comparison of regions, developed status and income group, the scores for vulnerability, sensitivity, exposure and adaptive capacity were weighted by population size and the mean taken so that smaller nations did not contribute disproportionately to the overall regional mean.

The minimal model was run, as described above, with projected changes in climate and population growth, and also with either the climate or the population growth projections held constant to explore the specific influence of each of these two parameters acting individually, through the exposure index, on overall vulnerability. A fully dynamic model where changes in the exposure feed into sensitivity and adaptive capacity scores would be unduly speculative at this stage, as there is no quantitative information to allow any informed prediction about how parameters such as governance or economy are likely to change for each nation, positively or negatively, in response to climatic or population change. To estimate the potential scale of the effects of likely changes in these indices on vulnerability, a sensitivity analysis was conducted, where simulations were run in which sensitivity and adaptive capacity were varied systematically. In this analysis, for each nation, sensitivity and adaptive capacity scores were varied from −100% to +100% of their values in the main model at 10% increments. The vulnerability model was run for all possible combinations of these sensitivity and adaptive capacity scores (*n* = 441). For each nation, the percentage change between the simulated vulnerability and that predicted in the main model was recorded. The median of the percentage change across all nations was then plotted against both the change in sensitivity and the change in adaptive capacity, to show the median change in vulnerability under each potential scenario with the projected changes in climate and population.

The database of indicators was built in Microsoft Excel 2010. All data analysis was performed using R (Pebesma & Bivand, 2005; Neuwirth, 2011; R Core Team, 2012; South *et al.*, 2012; Harrell, 2013) under the R studio interface version 0.97.449 (RStudio, 2012). The significance level was set at *P* = 0.05 for all tests. The ranking of nations in their vulnerability scores between models was compared using Wilcoxon signed-rank sum tests. Spearman rank correlation was used to determine the influence of each indicator and the sensitivity, exposure and adaptive capacity index scores on the vulnerability scores.

All indicator and index scores, World Bank region, developed status, income group and food-deficit status for the individual nations included in the final analysis can be found in the supplementary data available online (S1–S5).

## Results

### Model comparisons

There were no consistent differences in the results generated by the full model, including all 76 indicators, and the minimal additive model (*V* = 4577, *P* = 0.75, *N* = 144). Similarly, no significant differences were seen using either the additive or multiplicative models, in terms of the ranking of nations in sensitivity (*V* = 4767, *P* = 0.62, *N* = 148), exposure (*V* = 4746, *P* = 0.23, *N* = 148) or adaptive capacity (*V* = 4921.5, *P* = 0.98, *N* = 148). Overall vulnerability rankings also showed no significant difference between models [*V* = 4967, *P* = 0.61, *N* = 148 for Eqns (2) and (3)] as was found in previous work (Allison *et al.*, 2009). Hence, only the results from the additive minimal model are presented in detail here.

### Sensitivity

**Fig 1:**
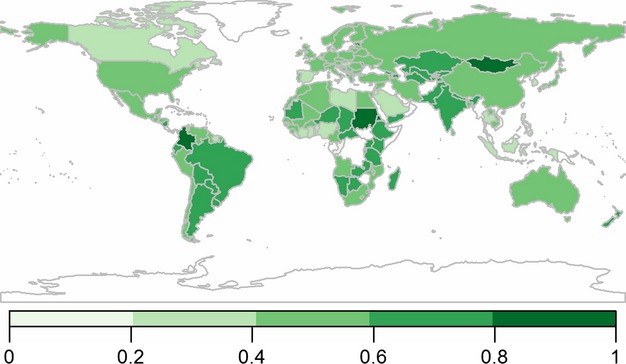
Sensitivity: nutritional reliance on home-produced grazing animal-based food products and level of food security. 0–1 = low to high sensitivity. Nations not included in the analysis are represented in white.

The sensitivity score, as calculated here, was composed of a nation's level of self-sufficiency with regard to grazing animal-based food products, the nutritional contribution of animal-based food products to diet and the level of food security (Table1). The global distribution of scores for sensitivity is shown in Fig.1. Within the top tenth percentile of sensitivity scores (Table2), which indicates the 15 most sensitive nations, six nations are located in Africa and a further five in Asia. No nations in this group are classified as high income or developed and five are classified as food-deficit nations (Table S1). Within the bottom tenth percentile, representing the least sensitive nations, there are four high-income and developed nations, while five food-deficit nations are also identified (Table2 and Table S1).

**Table 2:** The modelled rankings and (scores) of the 15 nations with highest and the 15 nations with lowest vulnerability (where the scores for lowest vulnerability = 0 and the highest = 1), representing the upper and lower tenth percentile of the data set for the minimal additive vulnerability model, where *n* = 148. Rankings and (scores) for sensitivity, exposure and adaptive capacity are also presented. In addition, the vulnerability estimated by the model when either climate or population change contributors to the exposure index are allowed to vary independently

Nation	Vulnerability rank (score)	Sensitivity rank (score)	Exposure rank (score)	Adaptive capacity rank (score)	Vulnerability (climate change only) Rank (score)	Vulnerability (population growth only) Rank (score)
Most vulnerable nations						
Kenya	1 (1.00)	7 (0.70)	1 (0.67)	125 (0.12)	1 (1.00)	4 (0.94)
Burundi	2 (0.93)	5 (0.75)	6 (0.42)	141 (0.03)	2 (0.95)	3 (0.96)
Eritrea	3 (0.91)	3 (0.89)	15 (0.39)	116 (0.17)	4 (0.88)	2 (1.00)
Sudan (former)	4 (0.87)	2 (0.89)	31 (0.32)	115 (0.17)	7 (0.83)	1 (1.00)
Swaziland	5 (0.85)	11 (0.64)	17 (0.37)	144 (0.01)	3 (0.90)	10 (0.86)
Mongolia	6 (0.81)	1 (1.00)	79 (0.20)	100 (0.26)	6 (0.87)	7 (0.89)
Zambia	7 (0.81)	22 (0.57)	20 (0.37)	143 (0.01)	8 (0.77)	6 (0.90)
Chad	8 (0.79)	27 (0.51)	10 (0.41)	142 (0.02)	13 (0.71)	5 (0.91)
Niger	9 (0.79)	43 (0.45)	2 (0.52)	132 (0.09)	15 (0.70)	13 (0.86)
United Republic of Tanzania	10 (0.78)	21 (0.58)	7 (0.42)	122 (0.13)	10 (0.72)	12 (0.86)
Uganda	11 (0.77)	35 (0.49)	5 (0.44)	133 (0.08)	18 (0.68)	9 (0.87)
Mauritania	12 (0.76)	36 (0.48)	3 (0.49)	119 (0.14)	11 (0.71)	21 (0.79)
Central African Republic	13 (0.76)	17 (0.61)	63 (0.23)	147 (0.00)	9 (0.75)	8 (0.89)
Ethiopia	14 (0.75)	18 (0.61)	23 (0.36)	118 (0.14)	16 (0.69)	11 (0.86)
Namibia	15 (0.74)	8 (0.66)	25 (0.35)	111 (0.21)	14 (0.71)	16 (0.82)
Least vulnerable nations						
New Zealand	134 (0.15)	45 (0.44)	98 (0.14)	5 (0.83)	137 (0.15)	132 (0.19)
Netherlands	135 (0.13)	49 (0.43)	115 (0.06)	11 (0.78)	132 (0.17)	135 (0.15)
Sweden	136 (0.11)	37 (0.47)	116 (0.06)	3 (0.85)	136 (0.16)	136 (0.13)
Republic of Korea	137 (0.11)	145 (0.10)	108 (0.09)	25 (0.51)	138 (0.14)	137 (0.13)
Belgium	138 (0.11)	110 (0.28)	122 (0.05)	18 (0.65)	134 (0.16)	138 (0.12)
United Kingdom	139 (0.11)	90 (0.33)	119 (0.05)	17 (0.70)	135 (0.16)	139 (0.12)
Switzerland	140 (0.10)	57 (0.42)	107 (0.09)	4 (0.85)	142 (0.12)	140 (0.12)
Finland	141 (0.08)	48 (0.43)	121 (0.05)	2 (0.86)	140 (0.13)	142 (0.09)
Norway	142 (0.07)	74 (0.36)	109 (0.08)	6 (0.83)	143 (0.10)	141 (0.09)
Germany	143 (0.06)	84 (0.34)	131 (0.01)	13 (0.77)	141 (0.12)	144 (0.06)
Canada	144 (0.06)	118 (0.26)	104 (0.12)	10 (0.79)	146 (0.06)	143 (0.08)
Denmark	145 (0.05)	77 (0.35)	123 (0.05)	7 (0.83)	144 (0.10)	145 (0.06)
Austria	146 (0.05)	106 (0.29)	118 (0.05)	12 (0.77)	145 (0.09)	146 (0.05)
United States of America	147 (0.00)	78 (0.35)	99 (0.13)	1 (1.00)	148 (0.00)	147 (0.01)
Japan	148 (0.00)	119 (0.25)	128 (0.02)	9 (0.80)	147 (0.06)	148 (0.00)

In terms of self-sufficiency and how reliant a nation is on its own livestock production, the 15 most sensitive nations include five African, five Asian and three South American nations (Tables S1 and S2). New Zealand is the only high-income and developed nation within the top tenth percentile. Four nations are classified as least developed and seven are considered food-deficit nations; five food-deficit nations are also within the lower tenth percentile. Twelve of the 15 least food secure nations are located in Sub-Saharan Africa, nine have a low income and nine are classified as food deficit (Tables S1 and S2). In contrast, eight of the top tenth percentile of nations for the contribution of grazing animal products to nutritional intake are located in Asia and five in Europe with only one low-income and one least developed nation present (Tables S1 and S2); three nations are classified food deficit. The lowest tenth percentile for this category comprises nine African and six Asian nations, all of which fall into the low or lower middle income groups with two exceptions. Only three nations are not classified as food-deficit nations.

South Asia ranks the most sensitive region (Table3), with developing and lower middle income nations also ranking the most sensitive. The least sensitive region is Europe and Central Asia.

**Table 3:** The rank of mean vulnerability, sensitivity, exposure and adaptive capacity scores, weighted by the nations' population size, presented for World Bank regions, income group and developed status of nations for the minimal additive vulnerability model. In addition, the vulnerability estimated by the model when either climate or population change contributors to the exposure index are allowed to vary independently. Number of nations in category = *n*

	*n*	Vulnerability (Rank)	Sensitivity (Rank)	Exposure (Rank)	Adaptive capacity (Rank)	Vulnerability (climate change only) (Rank)	Vulnerability (population growth only) (Rank)
World Bank regions							
East Asia and Pacific	17	2	3	3	3	2	2
Europe and Central Asia	47	7	7	7	6	7	7
Latin America and Caribbean	24	5	5	5	4	5	5
Middle East and North Africa	13	4	4	4	5	4	4
North America	2	3	2	2	2	3	3
South Asia	5	1	1	1	1	1	1
Sub-Saharan Africa	40	6	6	6	7	6	6
Income group							
Low	28	3	4	4	4	4	4
Lower middle	39	1	1	2	2	1	1
Upper middle	42	2	2	1	1	2	2
High	39	4	3	3	3	3	3
Developed status							
Least developed	36	2	3	3	3	2	2
Developing	77	1	1	1	1	1	1
Developed	35	3	2	2	2	3	3

### Exposure

**Fig 2:**
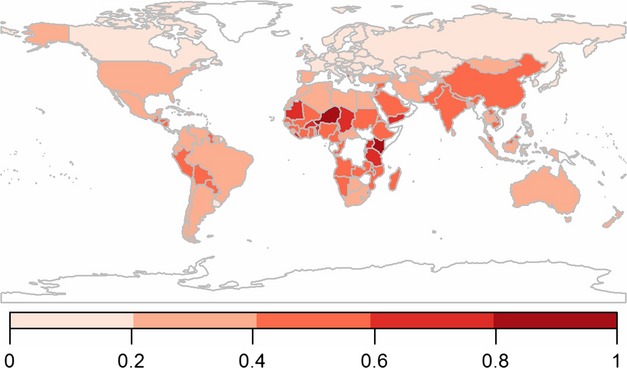
Exposure: impact of projected changes in climate based on the current percentage of population affected by drought, flooding and extreme weather and projected population growth of nations. 0–1 = low to high exposure. Nations not included in the analysis are represented in white.

Exposure was composed of the predicted impact of changes in weather in addition to the projected change in population (Table1). The global distribution of scores for exposure by individual nation is shown in Fig.2. Within the top tenth percentile of nations in the exposure index (Table2), only three fall within the top tenth percentile for both contributing indicators (Table S3). Twelve of the 15 nations have a low or lower middle income and 11 have a least developed status, are located in Sub-Saharan Africa and are classified as food deficit (Table2 and Table S1). Thirteen of the 15 nations within the bottom tenth percentile for exposure score are located in Europe and are projected to have a static or negative population growth (Table S3). No nations within this group are classified as having a low income, to be least developed or to be in food deficit (Table S1).

Within the upper tenth percentile for population growth, 12 least developed and three developing nations are identified, of which 12 are classified as food-deficit nations (Tables S1 and S3). Twelve nations are located in Sub-Saharan Africa. In contrast, population growth is projected to be static or negative in 18 nations, 15 of which are located in Europe and include no low-income or least developed nations.

The upper tenth percentile of nations to be impacted by climate change include no developed and nine food-deficit nations (Tables S1 and S3). Within the lowest tenth percentile, only two lower middle income nations and no low-income nations and one least developed nation are found; no nation is food deficit.

South Asia, upper middle and developing nations are predicted to be the most exposed (Table3) and Europe and Central Asia the least exposed region.

### Adaptive capacity

**Fig 3:**
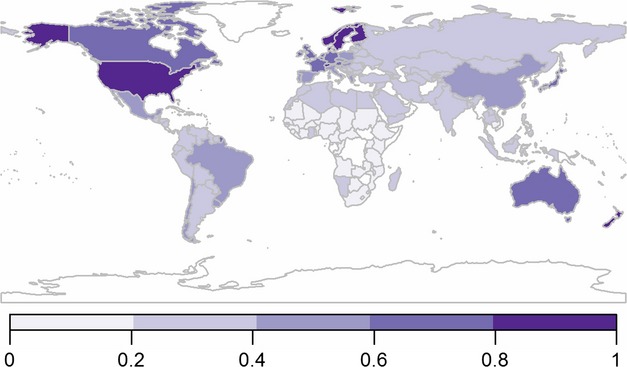
Adaptive capacity: a nation's ability to change in response to or cope with changes in climate and food demand based on health, economic and governance indicators. 0–1 = low to high adaptive capacity. Nations not included in the analysis are represented in white.

Adaptive capacity was composed of three indicators representing the health, economy and governance of nations (Table1). The global distribution of scores for adaptive capacity is shown in Fig.3. The top tenth percentile of nations, those with the highest adaptive capacity, are all high-income developed nations (Table2 and Table S1). In contrast, the lowest tenth percentile of nations is composed entirely of Sub-Saharan African nations and no high-income or developed nations; the lowest 34 ranking nations, in fact, are located in Sub-Saharan Africa (Table S4). Twelve of the 15 nations with the lowest adaptive capacity are considered to be least developed and a further 12 are classified as food deficit (Table2 and Table S1).

In the upper tenth percentile of the health index, all nations are classified as high income and developed (Tables S1 and S4), while in the lowest tenth percentile, all nations are located in Sub-Saharan Africa, 10 are classified as least developed, 10 have food-deficit classification and 12 have a low or lower middle income.

Within the top tenth percentile of the economy scores, 11 nations are classified as high income and developed (Tables S1 and S4). India, classified as a lower middle income nation, and three upper middle income nations are also identified. Five nations have a developing status; all other nations are developed. The lowest tenth percentile, those nations with the least wealth, includes nine Sub-Saharan African nations and no developed or high-income nations; seven nations are identified as food deficit.

All nations in the upper tenth percentile of the governance index are classified as high income and developed and include nations from Europe, North America and Australasia only (Tables S1 and S4). In contrast, none of these classifications are found in the lower tenth percentile, which includes seven food-deficit nations.

South Asia followed by North America are the regions shown to have greatest adaptive capacity (Table3). The middle-income and developing nations also rank highest for adaptive capacity. Sub-Saharan Africa is shown to have least adaptive capacity.

### Vulnerability

**Fig 4:**
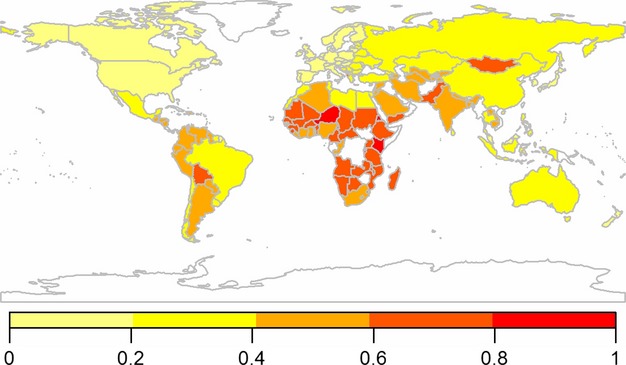
Overall vulnerability of nations to the impacts of population growth and climate change on grazing livestock and their contribution to food security. 0–1 = low to high vulnerability. Nations not included in the analysis are represented in white.

The global distribution of scores for the vulnerability index resulting from the combined minimal additive model is shown in Fig.4. All nations identified in the top tenth percentile of vulnerable nations are located in Sub-Saharan Africa, seven of which are in Eastern Africa (Table2). Only two nations have developing status and five have lower middle incomes; the remainder are low income and least developed; 11 nations are classified as food-deficit nations (Table S1). Within the least vulnerable tenth percentile, all nations are classified as developed and high income, with one exception; 11 nations are located in Europe (Tables2 and S1).

At the regional level, South Asia is most vulnerable (Table3) with the lower middle followed by the upper middle income groups, in addition to the developing nations. Europe and Central Asia, high-income and developed nations rank lowest.

In the model where only climate change within the exposure index was allowed to vary, 12 nations within the upper tenth percentile are located in Sub-Saharan Africa (Table2). As with the combined model, no high-income or developed nations are identified (Table S1); nine nations are classified as food deficit. The United States is ranked the least vulnerable nation, with 12 of the nations in the lower tenth percentile being both developed and high-income nations (Tables2 and S1).

In the model where only population growth was allowed to vary in the exposure index, within the top tenth percentile only Yemen is not located in Sub-Saharan Africa (Table2). Twelve food-deficit nations are identified and no high-income or developed nations (Table S1). The least vulnerable nation identified by the population growth model is Japan (Table2). Eleven European nations appear in the lower tenth percentile of nations resulting from the population growth model and all nations except one are classified high income and developed (Table2 and Table S1).

### Influence of indicators

Correlation (Spearman's rank) identifies self-sufficiency as the most influential parameter affecting sensitivity (*ρ* = 0.79, *P* < 0.01). Projected population growth is shown to have a highly significant influence on exposure score (*ρ* = 0.92, *P* < 0.01). Adaptive capacity is influenced most strongly by health (*ρ* = 0.97, *P* < 0.01) and governance (*ρ* = 0.75, *P* < 0.01). Adaptive capacity and exposure have the strongest correlations with vulnerability (*ρ* = 0.86 and *ρ* = 0.83 respectively, *P* < 0.01). When the influence of individual indicators on vulnerability is considered, there is a very strong influence of food security (*ρ* = 0.79, *P* < 0.01), health (*ρ* = 0.83, *P* < 0.01), governance (*ρ* = 0.73, *P* < 0.01) and both indicators of exposure (*ρ* = 0.74, *P* < 0.01 and *ρ* = 0.68, *P* < 0.01).

### Uncertainty in future sensitivity and adaptive capacity

**Fig 5:**
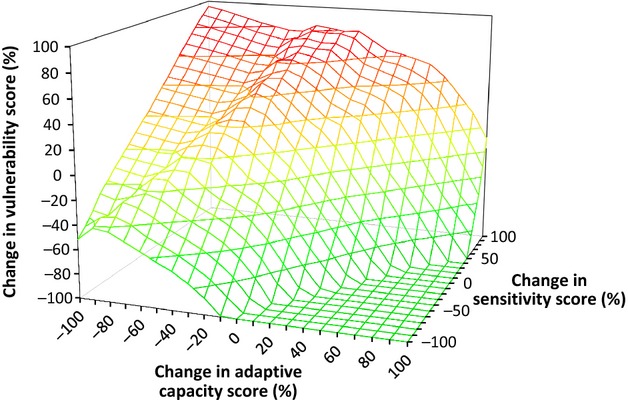
The percentage change in predicted vulnerability of nations to the impacts of population growth and climate change on grazing livestock and their contribution to food security, under potential future sensitivity and adaptive capacity scenarios, compared with vulnerability calculated on present values for sensitivity and adaptive capacity. Red = increase in vulnerability, green = decrease in vulnerability.

Changes in sensitivity and adaptive capacity, acting in concert with projected changes in climate and population, would be expected to impact substantially on vulnerability (Fig.5). As expected, nations with high sensitivity and low adaptive capacity are the most vulnerable, but changes in sensitivity have an almost linear impact on vulnerability, whereas increases in adaptive capacity result in a reduction in vulnerability of almost twice the amount. Reductions in adaptive capacity initially show the same trend by increasing vulnerability by twice the amount of the loss in adaptive capacity, but at greater adaptive capacity losses (more than 20%), smaller impacts on vulnerability are seen. Hence, with no change in sensitivity, a complete loss of adaptive capacity results in only a relatively small (30%) increase in vulnerability, compared to the scenario in which no change in adaptive capacity and a doubling in sensitivity increases vulnerability by almost 80% (Fig.5).

## Discussion

This analysis gives a broad overview of vulnerability, identifying nations and areas where livestock-based food production is most vulnerable to predicted population growth and climate change impacts. The model serves as a useful starting point for the identification of those nations at which policy, support and interventions might be focused. The results also provide a useful indicator of how strongly different economic, political and social characteristics effect vulnerability and, by consideration of the least vulnerable nations, which might safeguard against vulnerability.

While more developed and higher income nations incorporate animal-based products as an optional part of their diet, food derived from livestock represent a necessity for nations in which factors such as climate and geography render arable production unproductive in many areas (Kabubo-Mariara, 2009) and is reflected in the high number of Asian nations in the upper tenth percentile of nations for the contribution of grazing animal-based products to nutritional intake. The development and growing income of Asian nations might also be expected to drive an increase in their demand for animal products in the future (Steinfeld *et al.*, 2006), which may increase their vulnerability due to an increase in sensitivity, if no concurrent increase in adaptive capacity is seen.

An attempt was made here to explore the importance of the self-sufficiency of nations, with the intention of quantifying their dependence on their own grazing livestock. Previous work (Schmidhuber & Tubiello, 2007) has shown that self-sufficiency is neither necessary nor sufficient to guarantee food security; Singapore and Hong Kong, two areas where no agriculture exists, have food secure populations, while in contrast, India is self-sufficient but has a large proportion of its population that is not food secure. We also suggest that self-sufficiency has a strong influence on sensitivity. Nations which are currently self-sufficient are less likely to have the established infrastructure or trade networks to allow them to compensate rapidly for environmental changes which impact on their livestock production systems. The results presented show that African and some Asian nations are likely to be the most vulnerable, as their food security is already compromised and further constraints result from the current harsh climatic conditions and lack of economic and technical support. Population growth is likely to have a major impact on vulnerability as it results in both increased competition for resources and increased demands for food. The latter places further pressure on resources which are compromised by changes in climate. When population growth or climate was changed independently, it was notable that both strongly affected the same region of sub-Saharan Africa. Consequently, many African, particularly Sub-Saharan African nations, which are highly exposed to both population growth and temperature increase, score highly for the exposure index.

The minimal additive model used in this study allows a broad overview of relative vulnerability to be obtained with the identification of some important factors at the global scale. Future developments in this approach should include the incorporation of information on livestock stocking densities, changes in water availability, classification of production system and vegetation and disease epidemiology. Consideration of access to veterinary and extension services and enrolment in livestock insurance schemes (Skees & Enkh-Amgalan, 2002) and the level of education within a nation would also add refinement to the adaptive capacity index.

While the model includes projections for climate and population, which together comprise the exposure index, it then incorporates historical data relating to adaptive capacity and sensitivity. This approach is not ideal and a comprehensive simulation would also incorporate projections of future change in sensitivity and adaptive capacity and allow feedback between all the terms included, as both adaptive capacity and sensitivity may well change in relation to climate and population growth; this would give a valuable increase in sophistication. However, at present, insufficient quantitative data exist to predict the likely nature of such changes globally. For example, in some nations, growth in the amount of grazing animal-based food products has often been facilitated by favourable technical, economic and policy conditions (Thornton & Gerber, 2010), which supports an expected positive influence of governance on adaptive capacity, and in turn on vulnerability. Conversely, the introduction of climate change mitigation strategies may affect the production technologies and farming systems available and constrain future growth of the livestock sector (Thornton & Gerber, 2010). In contrast, impacts could also be reduced by investments in irrigation, food storage systems and increased food imports, in addition to a policy environment allowing freer trade and investment in transportation, communication and irrigation infrastructures (Schmidhuber & Tubiello, 2007). Given the level of uncertainty in these factors, it is not possible to accurately capture the interactions between these characteristics on a global scale at this stage. Hence, as an initial step, a sensitivity analysis was included which modelled the range of possible changes in adaptive capacity and sensitivity that might accompany projected changes in climate and population. The results presented show that, as expected, the predicted vulnerability might be reduced by improvements to adaptive capacity and reduction in sensitivity, but that increases in adaptive capacity appear to have a proportionately larger effect than reductions in sensitivity. Notably, the model suggests that governance is a particularly important factor influencing adaptive capacity and therefore, although the model indicates that policy, support and interventions should be targeted at the most vulnerable nations, any implementation will require considerable care in the management of structural reform.

## Supporting Information

Table S1 Information on the nations included in the analysis of vulnerability to human population growth and climate change on livestock-based food production. **Table S2.** Rank and score of nations for the sensitivity element of the vulnerability index of livestock-based food production scaled from zero to one. Bold indicates upper tenth percentile, italics indicates lower tenth percentiles. **Table S3.** Rank and scores for the impact of projected climate change and population growth for the exposure element of the vulnerability index of livestock-based food production scaled from zero to one. Bold indicates upper tenth percentile, italics indicates lower tenth percentiles. **Table S4.** Rank and scores for the adaptive capacity element of the vulnerability index of livestock-based food production scaled from zero to one. Bold indicates upper tenth percentile, italics indicates lower tenth percentiles. **Table S5.** Vulnerability, sensitivity, exposure and adaptive capacity rank and scores for all nations included in the vulnerability analysis of livestock-based food production, scaled from zero to one. Bold indicates upper tenth percentile, italics indicates lower tenth percentiles.
