# Supplementary material for: Livestock and food security: vulnerability to population growth and climate change
Source: Glob Chang Biol. 2014 May 2;20(10):3092–102. doi: 10.1111/gcb.12589 (PMC4282280; doi:10.1111/gcb.12589)
Supplement: Table S1 — Information on the nations included in the analysis of vulnerability to human population growth and climate change on livestock-based food production. Table S2. Rank and score of nations for the sensitivity element of the vulnerability index of livestock-based food production scaled from zero to one. Bold indicates upper tenth percentile, italics indicates lower tenth percentiles. Table S3. Rank and scores for the impact of projected climate change and population growth for the exposure element of the vulnerability index of livestock-based food production scaled from zero to one. Bold indicates upper tenth percentile, italics indicates lower tenth percentiles. Table S4. Rank and scores for the adaptive capacity element of the vulnerability index of livestock-based food production scaled from zero to one. Bold indicates upper tenth percentile, italics indicates lower tenth percentiles. Table S5. Vulnerability, sensitivity, exposure and adaptive capacity rank and scores for all nations included in the vulnerability analysis of livestock-based food production, scaled from zero to one. Bold indicates upper tenth percentile, italics indicates lower tenth percentiles. [file gcb0020-3092-SD1.docx]

Table S1. Information on the nations included in the analysis of vulnerability to human population growth and climate change on livestock-based food production.

| **Nation** | **α-3-code** | | **World Bank region** | **Income group** | **Developed status** | **Food-deficit** |
| --- | --- | --- | --- | --- | --- | --- |
| Albania | | ALB | Europe & Central Asia | Upper middle | Developing | No |
| Algeria | | DZA | Middle East & North Africa | Upper middle | Developing | No |
| Angola | | AGO | Sub-Saharan Africa | Upper middle | Least developed | No |
| Argentina | | ARG | Latin America & Caribbean | Upper middle | Developing | No |
| Armenia | | ARM | Europe & Central Asia | Lower middle | Developing | No |
| Australia | | AUS | East Asia & Pacific | High | Developed | No |
| Austria | | AUT | Europe & Central Asia | High | Developed | No |
| Azerbaijan | | AZE | Europe & Central Asia | Upper middle | Developing | No |
| Bangladesh | | BGD | South Asia | Low | Least developed | Yes |
| Belarus | | BLR | Europe & Central Asia | Upper middle | Developing | No |
| Belgium | | BEL | Europe & Central Asia | High | Developed | No |
| Belize | | BLZ | Latin America & Caribbean | Upper middle | Developing | No |
| Benin | | BEN | Sub-Saharan Africa | Low | Least developed | Yes |
| Bolivia (Plurinational State of) | | BOL | Latin America & Caribbean | Lower middle | Developing | No |
| Bosnia and Herzegovina | | BIH | Europe & Central Asia | Upper middle | Developing | No |
| Botswana | | BWA | Sub-Saharan Africa | Upper middle | Developing | No |
| Brazil | | BRA | Latin America & Caribbean | Upper middle | Developing | No |
| Bulgaria | | BGR | Europe & Central Asia | Upper middle | Developing | No |
| Burkina Faso | | BFA | Sub-Saharan Africa | Low | Least developed | Yes |
| Burundi | | BDI | Sub-Saharan Africa | Low | Least developed | Yes |
| Côte d'Ivoire | | CIV | Sub-Saharan Africa | Lower middle | Developing | Yes |
| Cambodia | | KHM | East Asia & Pacific | Low | Least developed | Yes |
| Cameroon | | CMR | Sub-Saharan Africa | Lower middle | Developing | Yes |
| Canada | | CAN | North America | High | Developed | No |
| Cape Verde | | CPV | Sub-Saharan Africa | Lower middle | Developing | No |
| Central African Republic | | CAF | Sub-Saharan Africa | Low | Least developed | Yes |
| Chad | | TCD | Sub-Saharan Africa | Low | Least developed | Yes |
| Chile | | CHL | Latin America & Caribbean | High | Developing | No |
| China | | CHN | East Asia & Pacific | Upper middle | Developing | No |
| Colombia | | COL | Latin America & Caribbean | Upper middle | Developing | No |
| Congo | | COG | Sub-Saharan Africa | Lower middle | Developing | Yes |
| Croatia | | HRV | Europe & Central Asia | High | Developed | No |
| Cyprus | | CYP | Europe & Central Asia | High | Developed | No |
| Czech Republic | | CZE | Europe & Central Asia | High | Developed | No |
| Denmark | | DNK | Europe & Central Asia | High | Developed | No |
| Dominican Republic | | DOM | Latin America & Caribbean | Upper middle | Developing | No |
| Ecuador | | ECU | Latin America & Caribbean | Upper middle | Developing | No |
| Egypt | | EGY | Middle East & North Africa | Lower middle | Developing | Yes |
| El Salvador | | SLV | Latin America & Caribbean | Lower middle | Developing | No |
| Eritrea | | ERI | Sub-Saharan Africa | Low | Least developed | Yes |
| Estonia | | EST | Europe & Central Asia | High | Developed | No |
| Ethiopia | | ETH | Sub-Saharan Africa | Low | Least developed | Yes |
| Fiji | | FJI | East Asia & Pacific | Upper middle | Developing | No |
| Finland | | FIN | Europe & Central Asia | High | Developed | No |
| France | | FRA | Europe & Central Asia | High | Developed | No |
| Gambia | | GMB | Sub-Saharan Africa | Low | Least developed | Yes |
| Georgia | | GEO | Europe & Central Asia | Lower middle | Developing | No |
| Germany | | DEU | Europe & Central Asia | High | Developed | No |
| Ghana | | GHA | Sub-Saharan Africa | Lower middle | Developing | Yes |
| Greece | | GRC | Europe & Central Asia | High | Developed | No |
| Guatemala | | GTM | Latin America & Caribbean | Lower middle | Developing | No |
| Guinea | | GIN | Sub-Saharan Africa | Low | Least developed | Yes |
| Guinea-Bissau | | GNB | Sub-Saharan Africa | Low | Least developed | Yes |
| Guyana | | GUY | Latin America & Caribbean | Lower middle | Developing | No |
| Haiti | | HTI | Latin America & Caribbean | Low | Least developed | Yes |
| Honduras | | HND | Latin America & Caribbean | Lower middle | Developing | Yes |
| Hungary | | HUN | Europe & Central Asia | Upper middle | Developed | No |
| India | | IND | South Asia | Lower middle | Developing | Yes |
| Indonesia | | IDN | East Asia & Pacific | Lower middle | Developing | Yes |
| Iran (Islamic Republic of) | | IRN | Middle East & North Africa | Upper middle | Developing | No |
| Ireland | | IRL | Europe & Central Asia | High | Developed | No |
| Israel | | ISR | Middle East & North Africa | High | Developed | No |
| Italy | | ITA | Europe & Central Asia | High | Developed | No |
| Jamaica | | JAM | Latin America & Caribbean | Upper middle | Developing | No |
| Japan | | JPN | East Asia & Pacific | High | Developed | No |
| Jordan | | JOR | Middle East & North Africa | Upper middle | Developing | No |
| Kazakhstan | | KAZ | Europe & Central Asia | Upper middle | Developing | No |
| Kenya | | KEN | Sub-Saharan Africa | Low | Developing | Yes |
| Kuwait | | KWT | Middle East & North Africa | High | Developed | No |
| Kyrgyzstan | | KGZ | Europe & Central Asia | Low | Developing | Yes |
| Lao People's Democratic Republic | | LAO | East Asia & Pacific | Lower middle | Least developed | Yes |
| Latvia | | LVA | Europe & Central Asia | High | Developing | No |
| Lebanon | | LBN | Middle East & North Africa | Upper middle | Developing | No |
| Lesotho | | LSO | Sub-Saharan Africa | Lower middle | Least developed | Yes |
| Liberia | | LBR | Sub-Saharan Africa | Low | Least developed | Yes |
| Libya | | LBY | Middle East & North Africa | Upper middle | Developing | No |
| Lithuania | | LTU | Europe & Central Asia | High | Developing | No |
| Luxembourg | | LUX | Europe & Central Asia | High | Developed | No |
| Madagascar | | MDG | Sub-Saharan Africa | Low | Developing | Yes |
| Malawi | | MWI | Sub-Saharan Africa | Low | Least developed | Yes |
| Malaysia | | MYS | East Asia & Pacific | Upper middle | Developing | No |
| Mali | | MLI | Sub-Saharan Africa | Low | Least developed | Yes |
| Mauritania | | MRT | Sub-Saharan Africa | Lower middle | Least developed | Yes |
| Mauritius | | MUS | Sub-Saharan Africa | Upper middle | Developing | No |
| Mexico | | MEX | Latin America & Caribbean | Upper middle | Developing | No |
| Mongolia | | MNG | East Asia & Pacific | Lower middle | Developing | Yes |
| Montenegro | | MNE | Europe & Central Asia | Upper middle | Developing | No |
| Morocco | | MAR | Middle East & North Africa | Lower middle | Developing | No |
| Mozambique | | MOZ | Sub-Saharan Africa | Low | Least developed | Yes |
| Namibia | | NAM | Sub-Saharan Africa | Upper middle | Developing | No |
| Nepal | | NPL | South Asia | Low | Least developed | Yes |
| Netherlands | | NLD | Europe & Central Asia | High | Developed | No |
| New Zealand | | NZL | East Asia & Pacific | High | Developed | No |
| Nicaragua | | NIC | Latin America & Caribbean | Lower middle | Developing | Yes |
| Niger | | NER | Sub-Saharan Africa | Low | Least developed | Yes |
| Nigeria | | NGA | Sub-Saharan Africa | Lower middle | Developing | Yes |
| Norway | | NOR | Europe & Central Asia | High | Developed | No |
| Pakistan | | PAK | South Asia | Lower middle | Developing | No |
| Panama | | PAN | Latin America & Caribbean | Upper middle | Developing | No |
| Paraguay | | PRY | Latin America & Caribbean | Lower middle | Developing | No |
| Peru | | PER | Latin America & Caribbean | Upper middle | Developing | No |
| Philippines | | PHL | East Asia & Pacific | Lower middle | Developing | Yes |
| Poland | | POL | Europe & Central Asia | High | Developed | No |
| Portugal | | PRT | Europe & Central Asia | High | Developed | No |
| Republic of Korea | | KOR | East Asia & Pacific | High | Developed | No |
| Republic of Moldova | | MDA | Europe & Central Asia | Lower middle | Developing | No |
| Romania | | ROU | Europe & Central Asia | Upper middle | Developing | No |
| Russian Federation | | RUS | Europe & Central Asia | High | Developing | No |
| Rwanda | | RWA | Sub-Saharan Africa | Low | Least developed | Yes |
| Saint Vincent and the Grenadines | | VCT | Latin America & Caribbean | Upper middle | Developing | No |
| Samoa | | WSM | East Asia & Pacific | Lower middle | Least developed | No |
| Saudi Arabia | | SAU | Middle East & North Africa | High | Developed | No |
| Senegal | | SEN | Sub-Saharan Africa | Lower middle | Least developed | Yes |
| Serbia | | SRB | Europe & Central Asia | Upper middle | Developing | No |
| Seychelles | | SYC | Sub-Saharan Africa | Upper middle | Developing | No |
| Sierra Leone | | SLE | Sub-Saharan Africa | Low | Least developed | Yes |
| Slovakia | | SVK | Europe & Central Asia | High | Developed | No |
| Slovenia | | SVN | Europe & Central Asia | High | Developed | No |
| Solomon Islands | | SLB | East Asia & Pacific | Lower middle | Least developed | Yes |
| South Africa | | ZAF | Sub-Saharan Africa | Upper middle | Developing | No |
| Spain | | ESP | Europe & Central Asia | High | Developed | No |
| Sri Lanka | | LKA | South Asia | Lower middle | Developing | Yes |
| Sudan (Former) | | SDN | Sub-Saharan Africa | Lower middle | Least developed | Yes |
| Suriname | | SUR | Latin America & Caribbean | Upper middle | Developing | No |
| Swaziland | | SWZ | Sub-Saharan Africa | Lower middle | Developing | No |
| Sweden | | SWE | Europe & Central Asia | High | Developed | No |
| Switzerland | | CHE | Europe & Central Asia | High | Developed | No |
| Syrian Arab Republic | | SYR | Middle East & North Africa | Lower middle | Developing | No |
| Tajikistan | | TJK | Europe & Central Asia | Low | Developing | Yes |
| Thailand | | THA | East Asia & Pacific | Upper middle | Developing | No |
| The Former Yugoslav Republic of Macedonia | | MKD | Europe & Central Asia | Upper middle | Developing | No |
| Timor Leste | | TLS | East Asia & Pacific | Lower middle | Least developed | No |
| Togo | | TGO | Sub-Saharan Africa | Low | Least developed | Yes |
| Trinidad and Tobago | | TTO | Latin America & Caribbean | High | Developed | No |
| Tunisia | | TUN | Middle East & North Africa | Upper middle | Developing | No |
| Turkey | | TUR | Europe & Central Asia | Upper middle | Developing | No |
| Turkmenistan | | TKM | Europe & Central Asia | Upper middle | Developing | No |
| Uganda | | UGA | Sub-Saharan Africa | Low | Least developed | Yes |
| Ukraine | | UKR | Europe & Central Asia | Lower middle | Developing | No |
| United Kingdom | | GBR | Europe & Central Asia | High | Developed | No |
| United Republic of Tanzania | | TZA | Sub-Saharan Africa | Low | Least developed | Yes |
| United States of America | | USA | North America | High | Developed | No |
| Uruguay | | URY | Latin America & Caribbean | High | Developing | No |
| Uzbekistan | | UZB | Europe & Central Asia | Lower middle | Developing | Yes |
| Vanuatu | | VUT | East Asia & Pacific | Lower middle | Least developed | No |
| Venezuela (Bolivarian Republic of) | | VEN | Latin America & Caribbean | Upper middle | Developing | No |
| Yemen | | YEM | Middle East & North Africa | Lower middle | Least developed | Yes |
| Zambia | | ZMB | Sub-Saharan Africa | Lower middle | Least developed | Yes |

**Table S2.** Rank and score of nations for the sensitivity element of the vulnerability index of livestock-based food production scaled from zero to one. Bold indicates upper tenth percentile, italics indicates lower tenth percentiles.

|  | **Self-sufficiency** | | **Food insecurity** | | **Nutritional contribution** | | **Sensitivity** | |
| --- | --- | --- | --- | --- | --- | --- | --- | --- |
| **Nation** | **Rank** | **Score** | **Rank** | **Score** | **Rank** | **Score** | **Rank** | **Score** |
| Albania | 36 | 0.26 | 64 | 0.21 | **8** | **0.67** | **12** | **0.63** |
| Algeria | 87 | 0.24 | 102 | 0.09 | 58 | 0.35 | 87 | 0.34 |
| Angola | 81 | 0.24 | 25 | 0.47 | 118 | 0.11 | 53 | 0.43 |
| Argentina | **6** | **0.27** | 93 | 0.11 | **3** | **0.73** | 16 | 0.62 |
| Armenia | 46 | 0.26 | 91 | 0.12 | 13 | 0.59 | 26 | 0.52 |
| Australia | 37 | 0.26 | *112* | *0.00* | 20 | 0.53 | 58 | 0.41 |
| Austria | 95 | 0.23 | *112* | *0.00* | 51 | 0.37 | 106 | 0.29 |
| Azerbaijan | 51 | 0.26 | 111 | 0.06 | **10** | **0.62** | 30 | 0.51 |
| Bangladesh | 43 | 0.26 | 44 | 0.32 | 124 | 0.10 | 88 | 0.33 |
| Belarus | 21 | 0.27 | *112* | *0.00* | 59 | 0.35 | 103 | 0.29 |
| Belgium | *139* | *0.12* | *112* | *0.00* | 27 | 0.48 | 110 | 0.28 |
| Belize | *137* | *0.13* | 73 | 0.16 | 108 | 0.19 | 129 | 0.20 |
| Benin | 102 | 0.23 | 69 | 0.18 | *140* | *0.03* | *137* | *0.18* |
| Bolivia (Plurinational State of) | 33 | 0.27 | 27 | 0.46 | 45 | 0.40 | **15** | **0.63** |
| Bosnia and Herzegovina | 90 | 0.24 | 98 | 0.10 | 34 | 0.45 | 63 | 0.40 |
| Botswana | 127 | 0.19 | **12** | **0.54** | 37 | 0.42 | **9** | **0.64** |
| Brazil | 17 | 0.27 | 77 | 0.16 | 38 | 0.42 | 44 | 0.44 |
| Bulgaria | 80 | 0.25 | 63 | 0.21 | 85 | 0.27 | 73 | 0.36 |
| Burkina Faso | 40 | 0.26 | 35 | 0.37 | 121 | 0.11 | 71 | 0.37 |
| Burundi | 50 | 0.26 | **1** | **1.00** | *134* | *0.05* | **5** | **0.75** |
| Côte d'Ivoire | 131 | 0.18 | 42 | 0.33 | 129 | 0.09 | 111 | 0.28 |
| Cambodia | 68 | 0.25 | 37 | 0.36 | *137* | *0.04* | 93 | 0.31 |
| Cameroon | 60 | 0.26 | 48 | 0.28 | 115 | 0.13 | 91 | 0.33 |
| Canada | 125 | 0.19 | *112* | *0.00* | 53 | 0.37 | 118 | 0.26 |
| Cape Verde | 133 | 0.17 | 55 | 0.24 | 77 | 0.29 | 83 | 0.34 |
| Central African Republic | **14** | **0.27** | 17 | 0.50 | 65 | 0.33 | 17 | 0.61 |
| Chad | 23 | 0.27 | **15** | **0.52** | 111 | 0.17 | 27 | 0.51 |
| Chile | 69 | 0.25 | 93 | 0.11 | 68 | 0.32 | 85 | 0.34 |
| China | 38 | 0.26 | 58 | 0.23 | 132 | 0.07 | 114 | 0.26 |
| Colombia | **7** | **0.27** | **9** | **0.56** | 26 | 0.49 | **4** | **0.75** |
| Congo | *142* | *0.10* | 84 | 0.14 | 127 | 0.09 | *144* | *0.11* |
| Croatia | 89 | 0.24 | *112* | *0.00* | 48 | 0.38 | 102 | 0.29 |
| Cyprus | 86 | 0.24 | 53 | 0.25 | 61 | 0.34 | 47 | 0.43 |
| Czech Republic | 94 | 0.24 | *112* | *0.00* | 44 | 0.40 | 97 | 0.31 |
| Denmark | 79 | 0.25 | *112* | *0.00* | 29 | 0.46 | 77 | 0.35 |
| Dominican Republic | 53 | 0.26 | 45 | 0.32 | 91 | 0.25 | 51 | 0.43 |
| Ecuador | **5** | **0.27** | 33 | 0.38 | 43 | 0.41 | 20 | 0.58 |
| Egypt | 63 | 0.26 | *112* | *0.00* | 74 | 0.30 | 121 | 0.25 |
| El Salvador | 99 | 0.23 | 55 | 0.24 | 62 | 0.34 | 55 | 0.42 |
| Eritrea | **1** | **0.27** | **2** | **0.94** | 71 | 0.32 | **3** | **0.89** |
| Estonia | 64 | 0.26 | 97 | 0.10 | 17 | 0.57 | 33 | 0.49 |
| Ethiopia | **8** | **0.27** | **5** | **0.59** | 98 | 0.24 | 18 | 0.61 |
| Fiji | 116 | 0.21 | 99 | 0.09 | 81 | 0.28 | 112 | 0.27 |
| Finland | 73 | 0.25 | *112* | *0.00* | **14** | **0.58** | 48 | 0.43 |
| France | 74 | 0.25 | *112* | *0.00* | 19 | 0.54 | 60 | 0.41 |
| Gambia | *140* | *0.11* | 55 | 0.24 | 126 | 0.09 | *140* | *0.17* |
| Georgia | 44 | 0.26 | 26 | 0.46 | 41 | 0.41 | **13** | **0.63** |
| Germany | 101 | 0.23 | *112* | *0.00* | 30 | 0.46 | 84 | 0.34 |
| Ghana | 130 | 0.18 | 95 | 0.10 | *136* | *0.04* | *146* | *0.10* |
| Greece | 119 | 0.20 | *112* | *0.00* | 22 | 0.51 | 75 | 0.36 |
| Guatemala | 96 | 0.23 | 28 | 0.46 | 114 | 0.15 | 46 | 0.44 |
| Guinea | 67 | 0.25 | 50 | 0.27 | 131 | 0.07 | 108 | 0.28 |
| Guinea-Bissau | 57 | 0.26 | 59 | 0.23 | 130 | 0.08 | 116 | 0.26 |
| Guyana | 105 | 0.23 | 84 | 0.14 | 87 | 0.26 | 101 | 0.30 |
| Haiti | 112 | 0.22 | **4** | **0.67** | 117 | 0.12 | 24 | 0.55 |
| Honduras | 47 | 0.26 | 66 | 0.20 | 76 | 0.29 | 68 | 0.38 |
| Hungary | 110 | 0.22 | 112 | 0.00 | 89 | 0.26 | 130 | 0.20 |
| India | **2** | **0.27** | 38 | 0.35 | 73 | 0.30 | 32 | 0.49 |
| Indonesia | 109 | 0.22 | 61 | 0.22 | *145* | *0.01* | *136* | *0.18* |
| Iran (Islamic Republic of) | 59 | 0.26 | 89 | 0.12 | 72 | 0.31 | 86 | 0.34 |
| Ireland | 78 | 0.25 | *112* | *0.00* | 23 | 0.51 | 67 | 0.38 |
| Israel | 70 | 0.25 | *112* | *0.00* | 69 | 0.32 | 113 | 0.26 |
| Italy | 123 | 0.20 | *112* | *0.00* | 40 | 0.42 | 104 | 0.29 |
| Jamaica | 83 | 0.24 | 67 | 0.20 | 99 | 0.23 | 89 | 0.33 |
| Japan | 85 | 0.24 | 73 | 0.16 | 112 | 0.15 | 119 | 0.25 |
| Jordan | 128 | 0.19 | 100 | 0.09 | 78 | 0.28 | 120 | 0.25 |
| Kazakhstan | 49 | 0.26 | *112* | *0.00* | **6** | **0.68** | 29 | 0.51 |
| Kenya | 10 | 0.27 | **14** | **0.52** | 32 | 0.45 | **7** | **0.70** |
| Kuwait | *136* | *0.14* | *112* | *0.00* | 66 | 0.33 | 131 | 0.20 |
| Kyrgyzstan | 26 | 0.27 | 71 | 0.18 | **4** | **0.71** | **10** | **0.64** |
| Lao People's Democratic Republic | 76 | 0.25 | 23 | 0.47 | *135* | *0.05* | 65 | 0.39 |
| Latvia | 93 | 0.24 | 88 | 0.13 | 52 | 0.37 | 72 | 0.37 |
| Lebanon | 124 | 0.19 | 101 | 0.09 | 39 | 0.42 | 80 | 0.35 |
| Lesotho | 82 | 0.24 | 46 | 0.31 | 105 | 0.19 | 69 | 0.38 |
| Liberia | *135* | *0.15* | 23 | 0.47 | *147* | *0.00* | 105 | 0.29 |
| Libya | 129 | 0.18 | *112* | *0.00* | 79 | 0.28 | 133 | 0.19 |
| Lithuania | 104 | 0.23 | *112* | *0.00* | 46 | 0.40 | 100 | 0.30 |
| Luxembourg | *141* | *0.10* | *112* | *0.00* | **7** | **0.68** | 61 | 0.40 |
| Madagascar | 31 | 0.27 | 21 | 0.49 | 94 | 0.25 | 25 | 0.55 |
| Malawi | 66 | 0.25 | 34 | 0.38 | *144* | *0.01* | 96 | 0.31 |
| Malaysia | *146* | *0.03* | 102 | 0.09 | 133 | 0.06 | *148* | *0.00* |
| Mali | 25 | 0.27 | 78 | 0.16 | 57 | 0.36 | 62 | 0.40 |
| Mauritania | 91 | 0.24 | 72 | 0.17 | 24 | 0.50 | 36 | 0.48 |
| Mauritius | *145* | *0.05* | 86 | 0.14 | 92 | 0.25 | *139* | *0.17* |
| Mexico | 65 | 0.25 | 89 | 0.12 | 83 | 0.27 | 95 | 0.31 |
| Mongolia | 16 | 0.27 | 29 | 0.43 | **1** | **1.00** | **1** | **1.00** |
| Montenegro | 113 | 0.21 | 75 | 0.16 | 25 | 0.49 | 42 | 0.45 |
| Morocco | 56 | 0.26 | 91 | 0.12 | 107 | 0.19 | 117 | 0.26 |
| Mozambique | 75 | 0.25 | **7** | **0.57** | *148* | *0.00* | 54 | 0.42 |
| Namibia | 71 | 0.25 | **6** | **0.58** | 60 | 0.35 | **8** | **0.66** |
| Nepal | 24 | 0.27 | 43 | 0.32 | 97 | 0.24 | 50 | 0.43 |
| Netherlands | 111 | 0.22 | *112* | *0.00* | **11** | **0.61** | 49 | 0.43 |
| New Zealand | **13** | **0.27** | *112* | *0.00* | 16 | 0.58 | 45 | 0.44 |
| Nicaragua | 29 | 0.27 | 36 | 0.36 | 93 | 0.25 | 38 | 0.47 |
| Niger | 28 | 0.27 | 62 | 0.22 | 54 | 0.37 | 43 | 0.45 |
| Nigeria | 88 | 0.24 | 79 | 0.15 | *142* | *0.02* | *142* | *0.16* |
| Norway | 35 | 0.26 | *112* | *0.00* | 31 | 0.45 | 74 | 0.36 |
| Pakistan | **9** | **0.27** | 39 | 0.34 | **9** | **0.64** | **6** | **0.71** |
| Panama | 72 | 0.25 | 52 | 0.25 | 55 | 0.37 | 40 | 0.46 |
| Paraguay | 30 | 0.27 | 31 | 0.40 | 103 | 0.21 | 41 | 0.46 |
| Peru | 52 | 0.26 | 50 | 0.27 | 109 | 0.18 | 76 | 0.36 |
| Philippines | *138* | *0.12* | 47 | 0.29 | *139* | *0.03* | *138* | *0.18* |
| Poland | 45 | 0.26 | *112* | *0.00* | 63 | 0.34 | 107 | 0.28 |
| Portugal | 114 | 0.21 | *112* | *0.00* | 67 | 0.32 | 124 | 0.24 |
| Republic of Korea | 107 | 0.23 | *112* | *0.00* | 122 | 0.10 | *145* | *0.10* |
| Republic of Moldova | 41 | 0.26 | 32 | 0.39 | 75 | 0.30 | 28 | 0.51 |
| Romania | 61 | 0.26 | *112* | *0.00* | 21 | 0.51 | 64 | 0.39 |
| Russian Federation | 58 | 0.26 | 109 | 0.07 | 28 | 0.48 | 56 | 0.42 |
| Rwanda | 22 | 0.27 | 22 | 0.49 | 116 | 0.13 | 39 | 0.46 |
| Saint Vincent and the Grenadines | *143* | *0.09* | 83 | 0.14 | 100 | 0.23 | *134* | *0.19* |
| Samoa | *144* | *0.08* | 104 | 0.09 | 106 | 0.19 | *143* | *0.13* |
| Saudi Arabia | 126 | 0.19 | 107 | 0.07 | 90 | 0.25 | 125 | 0.23 |
| Senegal | 106 | 0.23 | 40 | 0.34 | 110 | 0.17 | 70 | 0.38 |
| Serbia | 42 | 0.26 | 75 | 0.16 | 56 | 0.37 | 59 | 0.41 |
| Seychelles | 148 | 0.00 | 68 | 0.19 | 120 | 0.11 | *147* | *0.09* |
| Sierra Leone | 62 | 0.26 | 20 | 0.49 | *146* | *0.01* | 66 | 0.38 |
| Slovakia | 115 | 0.21 | 80 | 0.15 | 82 | 0.27 | 99 | 0.31 |
| Slovenia | 121 | 0.20 | *112* | *0.00* | 33 | 0.45 | 94 | 0.31 |
| Solomon Islands | *134* | *0.15* | 54 | 0.25 | *138* | *0.04* | *141* | *0.17* |
| South Africa | 34 | 0.26 | 105 | 0.08 | 95 | 0.25 | 109 | 0.28 |
| Spain | 108 | 0.23 | *112* | *0.00* | 86 | 0.27 | 128 | 0.21 |
| Sri Lanka | 117 | 0.21 | 30 | 0.41 | 128 | 0.09 | 81 | 0.35 |
| Sudan (Former) | 20 | 0.27 | 10 | 0.56 | **5** | **0.70** | **2** | **0.89** |
| Suriname | 132 | 0.18 | 49 | 0.28 | 119 | 0.11 | 115 | 0.26 |
| Swaziland | 100 | 0.23 | **12** | **0.54** | 49 | 0.38 | **11** | **0.64** |
| Sweden | 98 | 0.23 | 110 | 0.07 | **12** | **0.60** | 37 | 0.47 |
| Switzerland | 55 | 0.26 | *112* | *0.00* | 18 | 0.54 | 57 | 0.42 |
| Syrian Arab Republic | 48 | 0.26 | 108 | 0.07 | 64 | 0.33 | 92 | 0.32 |
| Tajikistan | **15** | **0.27** | **11** | **0.54** | 80 | 0.28 | 19 | 0.60 |
| Thailand | 97 | 0.23 | 65 | 0.20 | *141* | *0.03* | 132 | 0.19 |
| The Former Yugoslav Republic of Macedonia | 77 | 0.25 | 87 | 0.13 | 70 | 0.32 | 82 | 0.34 |
| Timor Leste | 122 | 0.20 | 16 | 0.52 | 104 | 0.20 | 34 | 0.49 |
| Togo | 118 | 0.20 | 41 | 0.33 | *143* | *0.01* | 122 | 0.25 |
| Trinidad and Tobago | *147* | *0.03* | 60 | 0.23 | 88 | 0.26 | 127 | 0.22 |
| Tunisia | 39 | 0.26 | *112* | *0.00* | 96 | 0.25 | 126 | 0.22 |
| Turkey | 27 | 0.27 | *112* | *0.00* | 84 | 0.27 | 123 | 0.24 |
| Turkmenistan | **3** | **0.27** | 96 | 0.10 | **2** | **0.76** | **14** | **0.63** |
| Uganda | 11 | 0.27 | 19 | 0.50 | 113 | 0.15 | 35 | 0.49 |
| Ukraine | 12 | 0.27 | *112* | *0.00* | 50 | 0.38 | 98 | 0.31 |
| United Kingdom | 92 | 0.24 | *112* | *0.00* | 36 | 0.43 | 90 | 0.33 |
| United Republic of Tanzania | 19 | 0.27 | **8** | **0.57** | 101 | 0.22 | 21 | 0.58 |
| United States of America | 32 | 0.27 | *112* | *0.00* | 35 | 0.44 | 78 | 0.35 |
| Uruguay | 18 | 0.27 | 81 | 0.15 | 42 | 0.41 | 52 | 0.43 |
| Uzbekistan | **4** | **0.27** | 70 | 0.18 | **15** | **0.58** | 23 | 0.56 |
| Vanuatu | 120 | 0.20 | 82 | 0.15 | 123 | 0.10 | *135* | *0.18* |
| Venezuela (Bolivarian Republic of) | 84 | 0.24 | 105 | 0.08 | 47 | 0.38 | 79 | 0.35 |
| Yemen | 103 | 0.23 | 18 | 0.50 | 102 | 0.21 | 31 | 0.50 |
| Zambia | 54 | 0.26 | **3** | **0.68** | 125 | 0.09 | 22 | 0.57 |

**Table S3.** Rank and scores for the impact of projected climate change and population growth for the exposure element of the vulnerability index of livestock-based food production scaled from zero to one. Bold indicates upper tenth percentile, italics indicates lower tenth percentiles.

| **Nation** | **Impact of projected**  **climate change** | | **Projected**  **population growth** | | **Exposure** | |
| --- | --- | --- | --- | --- | --- | --- |
|  | **Rank** | **Score** | **Rank** | **Score** | **Rank** | **Score** |
| Albania | **1** | **0.58** | *131* | *0.00* | **4** | **0.47** |
| Algeria | 106 | 0.00 | 64 | 0.24 | 77 | 0.20 |
| Angola | 74 | 0.01 | **6** | **0.44** | 21 | 0.36 |
| Argentina | 116 | 0.00 | 91 | 0.15 | 101 | 0.13 |
| Armenia | 54 | 0.02 | *131* | *0.00* | 129 | 0.02 |
| Australia | 58 | 0.02 | 80 | 0.18 | 86 | 0.17 |
| Austria | 103 | 0.00 | 114 | 0.06 | 118 | 0.05 |
| Azerbaijan | 45 | 0.04 | 88 | 0.17 | 85 | 0.17 |
| Bangladesh | 64 | 0.01 | 64 | 0.24 | 75 | 0.21 |
| Belarus | 101 | 0.00 | *131* | *0.00* | *141* | *0.00* |
| Belgium | 132 | 0.00 | 114 | 0.06 | 122 | 0.05 |
| Belize | 26 | 0.07 | **9** | **0.42** | **12** | **0.40** |
| Benin | 32 | 0.05 | **6** | **0.44** | **13** | **0.40** |
| Bolivia (Plurinational State of) | 44 | 0.04 | 45 | 0.28 | 52 | 0.26 |
| Bosnia and Herzegovina | 47 | 0.04 | *131* | *0.00* | 126 | 0.03 |
| Botswana | 38 | 0.04 | 52 | 0.27 | 55 | 0.25 |
| Brazil | 69 | 0.01 | 80 | 0.18 | 90 | 0.16 |
| Bulgaria | 108 | 0.00 | *131* | *0.00* | *144* | *0.00* |
| Burkina Faso | 18 | 0.11 | **13** | **0.39** | **9** | **0.41** |
| Burundi | **5** | **0.24** | 45 | 0.28 | **6** | **0.42** |
| Côte d'Ivoire | *135* | *0.00* | 36 | 0.32 | 48 | 0.26 |
| Cambodia | 40 | 0.04 | 45 | 0.28 | 49 | 0.26 |
| Cameroon | 95 | 0.00 | 32 | 0.34 | 45 | 0.28 |
| Canada | 107 | 0.00 | 95 | 0.14 | 104 | 0.12 |
| Cape Verde | 127 | 0.00 | 57 | 0.25 | 74 | 0.21 |
| Central African Republic | 91 | 0.00 | 45 | 0.28 | 63 | 0.23 |
| Chad | 28 | 0.06 | **6** | **0.44** | **10** | **0.41** |
| Chile | 73 | 0.01 | 80 | 0.18 | 91 | 0.16 |
| China | **4** | **0.24** | 101 | 0.11 | 44 | 0.29 |
| Colombia | 39 | 0.04 | 64 | 0.24 | 64 | 0.23 |
| Congo | 66 | 0.01 | 23 | 0.37 | 36 | 0.31 |
| Croatia | 121 | 0.00 | *131* | *0.00* | *145* | *0.00* |
| Cyprus | 120 | 0.00 | 57 | 0.25 | 72 | 0.21 |
| Czech Republic | 102 | 0.00 | 126 | 0.01 | 130 | 0.01 |
| Denmark | *138* | *0.00* | 114 | 0.06 | 123 | 0.05 |
| Dominican Republic | 89 | 0.00 | 57 | 0.25 | 70 | 0.21 |
| Ecuador | 33 | 0.05 | 64 | 0.24 | 62 | 0.23 |
| Egypt | 123 | 0.00 | 57 | 0.25 | 73 | 0.21 |
| El Salvador | 42 | 0.04 | 105 | 0.10 | 106 | 0.11 |
| Eritrea | **13** | **0.12** | 24 | 0.35 | **15** | **0.39** |
| Estonia | *138* | *0.00* | *131* | *0.00* | *146* | *0.00* |
| Ethiopia | 29 | 0.06 | 17 | 0.38 | 23 | 0.36 |
| Fiji | 17 | 0.11 | 101 | 0.11 | 83 | 0.18 |
| Finland | 130 | 0.00 | 114 | 0.06 | 121 | 0.05 |
| France | 119 | 0.00 | 109 | 0.07 | 113 | 0.06 |
| Gambia | 77 | 0.01 | **11** | **0.41** | 27 | 0.34 |
| Georgia | 46 | 0.04 | *131* | *0.00* | 125 | 0.03 |
| Germany | 118 | 0.00 | 126 | 0.01 | 131 | 0.01 |
| Ghana | 43 | 0.04 | 24 | 0.35 | 32 | 0.32 |
| Greece | 114 | 0.00 | 109 | 0.07 | 112 | 0.06 |
| Guatemala | 16 | 0.11 | 32 | 0.34 | 18 | 0.37 |
| Guinea | 93 | 0.00 | 17 | 0.38 | 34 | 0.31 |
| Guinea-Bissau | 61 | 0.02 | 45 | 0.28 | 59 | 0.24 |
| Guyana | **3** | **0.36** | 123 | 0.03 | 33 | 0.32 |
| Haiti | 51 | 0.03 | 64 | 0.24 | 68 | 0.22 |
| Honduras | **14** | **0.12** | 39 | 0.31 | 24 | 0.35 |
| Hungary | 90 | 0.00 | *131* | *0.00* | *139* | *0.00* |
| India | 19 | 0.10 | 57 | 0.25 | 40 | 0.29 |
| Indonesia | 82 | 0.00 | 80 | 0.18 | 93 | 0.15 |
| Iran (Islamic Republic of) | 23 | 0.09 | 73 | 0.21 | 57 | 0.25 |
| Ireland | 126 | 0.00 | 88 | 0.17 | 97 | 0.14 |
| Israel | *134* | *0.00* | 24 | 0.35 | 42 | 0.29 |
| Italy | 115 | 0.00 | 122 | 0.04 | 124 | 0.03 |
| Jamaica | 50 | 0.03 | 101 | 0.11 | 103 | 0.12 |
| Japan | 117 | 0.00 | 123 | 0.03 | 128 | 0.02 |
| Jordan | 78 | 0.01 | **3** | **0.45** | 16 | 0.37 |
| Kazakhstan | 81 | 0.00 | *131* | *0.00* | *137* | *0.00* |
| Kenya | **2** | **0.44** | 17 | 0.38 | **1** | **0.67** |
| Kuwait | *137* | *0.00* | 77 | 0.20 | 87 | 0.16 |
| Kyrgyzstan | 86 | 0.00 | 95 | 0.14 | 102 | 0.12 |
| Lao People's Democratic Republic | 49 | 0.03 | 45 | 0.28 | 53 | 0.26 |
| Latvia | *138* | *0.00* | *131* | *0.00* | *146* | *0.00* |
| Lebanon | 97 | 0.00 | 57 | 0.25 | 71 | 0.21 |
| Lesotho | 37 | 0.04 | 77 | 0.20 | 78 | 0.20 |
| Liberia | 52 | 0.03 | **3** | **0.45** | **14** | **0.39** |
| Libya | *138* | *0.00* | 52 | 0.27 | 69 | 0.22 |
| Lithuania | *138* | *0.00* | *131* | *0.00* | *146* | *0.00* |
| Luxembourg | *138* | *0.00* | 77 | 0.20 | 88 | 0.16 |
| Madagascar | 59 | 0.02 | **9** | **0.42** | 22 | 0.36 |
| Malawi | **8** | **0.18** | 36 | 0.32 | **8** | **0.41** |
| Malaysia | 94 | 0.00 | 39 | 0.31 | 54 | 0.25 |
| Mali | 36 | 0.04 | **11** | **0.41** | 19 | 0.37 |
| Mauritania | **7** | **0.21** | **13** | **0.39** | **3** | **0.49** |
| Mauritius | *138* | *0.00* | 95 | 0.14 | 105 | 0.11 |
| Mexico | 63 | 0.01 | 73 | 0.21 | 82 | 0.18 |
| Mongolia | 24 | 0.08 | 91 | 0.15 | 79 | 0.20 |
| Montenegro | 100 | 0.00 | 123 | 0.03 | 127 | 0.02 |
| Morocco | 88 | 0.00 | 80 | 0.18 | 95 | 0.15 |
| Mozambique | 62 | 0.02 | 17 | 0.38 | 30 | 0.32 |
| Namibia | 22 | 0.09 | 32 | 0.34 | 25 | 0.35 |
| Nepal | 31 | 0.05 | 36 | 0.32 | 37 | 0.31 |
| Netherlands | 128 | 0.00 | 109 | 0.07 | 115 | 0.06 |
| New Zealand | 131 | 0.00 | 88 | 0.17 | 98 | 0.14 |
| Nicaragua | 25 | 0.08 | 64 | 0.24 | 51 | 0.26 |
| Niger | **10** | **0.16** | **2** | **0.48** | **2** | **0.52** |
| Nigeria | 99 | 0.00 | 32 | 0.34 | 46 | 0.28 |
| Norway | 110 | 0.00 | 105 | 0.10 | 109 | 0.08 |
| Pakistan | 60 | 0.02 | 39 | 0.31 | 47 | 0.27 |
| Panama | 68 | 0.01 | 52 | 0.27 | 65 | 0.23 |
| Paraguay | 55 | 0.02 | 42 | 0.30 | 50 | 0.26 |
| Peru | **11** | **0.14** | 73 | 0.21 | 43 | 0.29 |
| Philippines | 98 | 0.00 | 42 | 0.30 | 60 | 0.24 |
| Poland | 104 | 0.00 | *131* | *0.00* | *142* | *0.00* |
| Portugal | 124 | 0.00 | 114 | 0.06 | 120 | 0.05 |
| Republic of Korea | 75 | 0.01 | 105 | 0.10 | 108 | 0.09 |
| Republic of Moldova | 67 | 0.01 | *131* | *0.00* | *135* | *0.01* |
| Romania | 83 | 0.00 | *131* | *0.00* | *138* | *0.00* |
| Russian Federation | 71 | 0.01 | *131* | *0.00* | *136* | *0.01* |
| Rwanda | **12** | **0.14** | 45 | 0.28 | 26 | 0.34 |
| Saint Vincent and the Grenadines | *138* | *0.00* | 126 | 0.01 | 133 | 0.01 |
| Samoa | *138* | *0.00* | 108 | 0.08 | 111 | 0.07 |
| Saudi Arabia | 129 | 0.00 | 17 | 0.38 | 35 | 0.31 |
| Senegal | 53 | 0.03 | 17 | 0.38 | 28 | 0.33 |
| Serbia | 105 | 0.00 | *131* | *0.00* | *143* | *0.00* |
| Seychelles | 84 | 0.00 | 91 | 0.15 | 100 | 0.13 |
| Sierra Leone | 87 | 0.00 | 52 | 0.27 | 67 | 0.22 |
| Slovakia | 133 | 0.00 | 126 | 0.01 | 132 | 0.01 |
| Slovenia | *138* | *0.00* | 126 | 0.01 | 133 | 0.01 |
| Solomon Islands | 76 | 0.01 | **13** | **0.39** | 29 | 0.33 |
| South Africa | 35 | 0.04 | 57 | 0.25 | 58 | 0.24 |
| Spain | 34 | 0.05 | 99 | 0.13 | 96 | 0.14 |
| Sri Lanka | **9** | **0.18** | 99 | 0.13 | 56 | 0.25 |
| Sudan (Former) | 41 | 0.04 | 24 | 0.35 | 31 | 0.32 |
| Suriname | 79 | 0.01 | 80 | 0.18 | 92 | 0.15 |
| Swaziland | **6** | **0.23** | 70 | 0.23 | 17 | 0.37 |
| Sweden | *138* | *0.00* | 109 | 0.07 | 116 | 0.06 |
| Switzerland | 122 | 0.00 | 101 | 0.11 | 107 | 0.09 |
| Syrian Arab Republic | 56 | 0.02 | 24 | 0.35 | 38 | 0.31 |
| Tajikistan | 27 | 0.07 | 80 | 0.18 | 76 | 0.21 |
| Thailand | 30 | 0.06 | 95 | 0.14 | 89 | 0.16 |
| The Former Yugoslav Republic of Macedonia | 48 | 0.03 | 114 | 0.06 | 110 | 0.07 |
| Timor Leste | 109 | 0.00 | 42 | 0.30 | 61 | 0.24 |
| Togo | 57 | 0.02 | 24 | 0.35 | 39 | 0.30 |
| Trinidad and Tobago | 125 | 0.00 | 109 | 0.07 | 114 | 0.06 |
| Tunisia | 85 | 0.00 | 80 | 0.18 | 94 | 0.15 |
| Turkey | 70 | 0.01 | 73 | 0.21 | 84 | 0.18 |
| Turkmenistan | *136* | *0.00* | 70 | 0.23 | 81 | 0.18 |
| Uganda | 21 | 0.09 | **3** | **0.45** | **5** | **0.44** |
| Ukraine | 92 | 0.00 | *131* | *0.00* | *140* | *0.00* |
| United Kingdom | 113 | 0.00 | 114 | 0.06 | 119 | 0.05 |
| United Republic of Tanzania | **15** | **0.12** | **13** | **0.39** | **7** | **0.42** |
| United States of America | 72 | 0.01 | 91 | 0.15 | 99 | 0.13 |
| Uruguay | 65 | 0.01 | 114 | 0.06 | 117 | 0.06 |
| Uzbekistan | 111 | 0.00 | 70 | 0.23 | 80 | 0.18 |
| Vanuatu | 112 | 0.00 | 24 | 0.35 | 41 | 0.29 |
| Venezuela (Bolivarian Republic of) | 80 | 0.01 | 52 | 0.27 | 66 | 0.22 |
| Yemen | 96 | 0.00 | **1** | **0.49** | **11** | **0.40** |
| Zambia | 20 | 0.10 | 24 | 0.35 | 20 | 0.37 |

**Table S4.** Rank and scores for the adaptive capacity element of the vulnerability index of livestock-based food production scaled from zero to one. Bold indicates upper tenth percentile, italics indicates lower tenth percentiles.

|  | **Health (life expectancy)** | | **Economy (GDP)** | | **Governance** | | **Adaptive Capacity** | |
| --- | --- | --- | --- | --- | --- | --- | --- | --- |
| **Nation** | **Rank** | **Score** | **Rank** | **Score** | **Rank** | **Score** | **Rank** | **Score** |
| Albania | 29 | 0.83 | 102 | 0.00 | 65 | 0.01 | 42 | 0.37 |
| Algeria | 72 | 0.71 | 46 | 0.01 | 114 | 0.00 | 76 | 0.32 |
| Angola | *139* | *0.09* | 56 | 0.01 | 133 | 0.00 | *139* | *0.04* |
| Argentina | 37 | 0.79 | 28 | 0.03 | 75 | 0.00 | 45 | 0.37 |
| Armenia | 54 | 0.74 | 110 | 0.00 | 74 | 0.00 | 68 | 0.33 |
| Australia | **4** | **0.96** | 13 | 0.08 | **11** | **0.65** | **14** | **0.76** |
| Austria | 18 | 0.92 | 27 | 0.03 | **8** | **0.77** | **12** | **0.77** |
| Azerbaijan | 84 | 0.65 | 63 | 0.00 | 118 | 0.00 | 88 | 0.29 |
| Bangladesh | 95 | 0.59 | 53 | 0.01 | 116 | 0.00 | 97 | 0.27 |
| Belarus | 85 | 0.64 | 62 | 0.00 | 128 | 0.00 | 90 | 0.29 |
| Belgium | 19 | 0.92 | 22 | 0.03 | **14** | **0.51** | 18 | 0.65 |
| Belize | 35 | 0.80 | *139* | *0.00* | 58 | 0.01 | 47 | 0.36 |
| Benin | 126 | 0.23 | 117 | 0.00 | 79 | 0.00 | 127 | 0.10 |
| Bolivia (Plurinational State of) | 105 | 0.53 | 91 | 0.00 | 96 | 0.00 | 106 | 0.24 |
| Bosnia and Herzegovina | 40 | 0.78 | 94 | 0.00 | 81 | 0.00 | 52 | 0.35 |
| Botswana | *134* | *0.16* | 98 | 0.00 | 34 | 0.14 | 121 | 0.13 |
| Brazil | 69 | 0.72 | **7** | **0.15** | 46 | 0.04 | 36 | 0.41 |
| Bulgaria | 59 | 0.73 | 67 | 0.00 | 44 | 0.05 | 55 | 0.35 |
| Burkina Faso | 128 | 0.21 | 111 | 0.00 | 69 | 0.01 | 129 | 0.10 |
| Burundi | *140* | *0.07* | *136* | *0.00* | *134* | *0.00* | *141* | *0.03* |
| Côte d'Ivoire | 129 | 0.21 | 84 | 0.00 | *138* | *0.00* | 130 | 0.09 |
| Cambodia | 111 | 0.42 | 104 | 0.00 | 121 | 0.00 | 112 | 0.19 |
| Cameroon | *137* | *0.10* | 87 | 0.00 | 119 | 0.00 | *137* | *0.05* |
| Canada | **11** | **0.93** | **10** | **0.11** | **10** | **0.73** | **10** | **0.79** |
| Cape Verde | 55 | 0.74 | *138* | *0.00* | 37 | 0.09 | 44 | 0.37 |
| Central African Republic | *146* | *0.01* | *137* | *0.00* | *142* | *0.00* | *147* | *0.00* |
| Chad | *142* | *0.05* | 114 | 0.00 | *145* | *0.00* | *142* | *0.02* |
| Chile | 26 | 0.88 | 40 | 0.02 | 18 | 0.39 | 19 | 0.57 |
| China | 66 | 0.72 | **2** | **0.41** | 103 | 0.00 | 26 | 0.51 |
| Colombia | 63 | 0.73 | 33 | 0.02 | 86 | 0.00 | 66 | 0.34 |
| Congo | 122 | 0.27 | 101 | 0.00 | 130 | 0.00 | 123 | 0.12 |
| Croatia | 31 | 0.81 | 61 | 0.00 | 38 | 0.08 | 38 | 0.40 |
| Cyprus | 23 | 0.89 | 83 | 0.00 | 21 | 0.31 | 21 | 0.54 |
| Czech Republic | 28 | 0.84 | 43 | 0.01 | 23 | 0.26 | 28 | 0.50 |
| Denmark | 24 | 0.89 | 31 | 0.02 | **2** | **0.93** | **7** | **0.83** |
| Dominican Republic | 68 | 0.72 | 65 | 0.00 | 88 | 0.00 | 74 | 0.33 |
| Ecuador | 38 | 0.78 | 57 | 0.00 | 115 | 0.00 | 51 | 0.35 |
| Egypt | 70 | 0.72 | 39 | 0.02 | 94 | 0.00 | 73 | 0.33 |
| El Salvador | 81 | 0.68 | 88 | 0.00 | 61 | 0.01 | 82 | 0.31 |
| Eritrea | 116 | 0.38 | *135* | *0.00* | *146* | *0.00* | 116 | 0.17 |
| Estonia | 39 | 0.78 | 92 | 0.00 | 20 | 0.33 | 27 | 0.50 |
| Ethiopia | 118 | 0.32 | 79 | 0.00 | 123 | 0.00 | 118 | 0.14 |
| Fiji | 91 | 0.61 | 130 | 0.00 | 110 | 0.00 | 94 | 0.27 |
| Finland | 21 | 0.91 | 35 | 0.02 | **1** | **1.00** | **2** | **0.86** |
| France | **8** | **0.95** | **5** | **0.18** | 16 | 0.44 | 16 | 0.70 |
| Gambia | 120 | 0.30 | *142* | *0.00* | 93 | 0.00 | 120 | 0.14 |
| Georgia | 64 | 0.73 | 103 | 0.00 | 55 | 0.01 | 69 | 0.33 |
| Germany | 20 | 0.91 | **4** | **0.23** | **13** | **0.58** | **13** | **0.77** |
| Ghana | 110 | 0.46 | 77 | 0.00 | 50 | 0.03 | 109 | 0.22 |
| Greece | 17 | 0.92 | 32 | 0.02 | 39 | 0.07 | 31 | 0.45 |
| Guatemala | 82 | 0.66 | 70 | 0.00 | 100 | 0.00 | 84 | 0.30 |
| Guinea | 131 | 0.18 | 125 | 0.00 | *141* | *0.00* | *135* | *0.08* |
| Guinea-Bissau | *145* | *0.01* | *144* | *0.00* | 129 | 0.00 | *146* | *0.00* |
| Guyana | 89 | 0.62 | 133 | 0.00 | 78 | 0.00 | 91 | 0.28 |
| Haiti | 114 | 0.40 | 116 | 0.00 | *140* | *0.00* | 114 | 0.18 |
| Honduras | 74 | 0.71 | 97 | 0.00 | 101 | 0.00 | 77 | 0.32 |
| Hungary | 48 | 0.75 | 51 | 0.01 | 31 | 0.17 | 34 | 0.41 |
| India | 107 | 0.50 | **9** | **0.12** | 85 | 0.00 | 92 | 0.28 |
| Indonesia | 93 | 0.60 | 18 | 0.05 | 91 | 0.00 | 86 | 0.29 |
| Iran (Islamic Republic of) | 75 | 0.71 | 24 | 0.03 | *137* | *0.00* | 70 | 0.33 |
| Ireland | **10** | **0.94** | 41 | 0.01 | **12** | **0.64** | **15** | **0.71** |
| Israel | **6** | **0.96** | 36 | 0.02 | 52 | 0.03 | 32 | 0.45 |
| Italy | **3** | **0.96** | **8** | **0.14** | 36 | 0.10 | 22 | 0.54 |
| Jamaica | 73 | 0.71 | 99 | 0.00 | 57 | 0.01 | 75 | 0.33 |
| Japan | **1** | **0.99** | **3** | **0.38** | 17 | 0.40 | **9** | **0.80** |
| Jordan | 65 | 0.72 | 81 | 0.00 | 59 | 0.01 | 71 | 0.33 |
| Kazakhstan | 98 | 0.58 | 48 | 0.01 | 95 | 0.00 | 98 | 0.27 |
| Kenya | 124 | 0.26 | 76 | 0.00 | 109 | 0.00 | 125 | 0.12 |
| Kuwait | 45 | 0.76 | 52 | 0.01 | 51 | 0.03 | 48 | 0.36 |
| Kyrgyzstan | 90 | 0.61 | 124 | 0.00 | 122 | 0.00 | 93 | 0.27 |
| Lao People's Democratic Republic | 103 | 0.55 | 115 | 0.00 | 127 | 0.00 | 104 | 0.25 |
| Latvia | 60 | 0.73 | 82 | 0.00 | 35 | 0.13 | 41 | 0.39 |
| Lebanon | 76 | 0.70 | 73 | 0.00 | 105 | 0.00 | 79 | 0.31 |
| Lesotho | *148* | *0.00* | *134* | *0.00* | 60 | 0.01 | *145* | *0.00* |
| Liberia | 125 | 0.25 | *140* | *0.00* | 113 | 0.00 | 126 | 0.11 |
| Libya | 42 | 0.77 | 59 | 0.00 | *139* | *0.00* | 61 | 0.34 |
| Lithuania | 67 | 0.72 | 75 | 0.00 | 32 | 0.16 | 39 | 0.40 |
| Luxembourg | **14** | **0.93** | 64 | 0.00 | **5** | **0.88** | **8** | **0.81** |
| Madagascar | 104 | 0.53 | 112 | 0.00 | 107 | 0.00 | 105 | 0.24 |
| Malawi | 133 | 0.17 | 123 | 0.00 | 72 | 0.01 | *134* | *0.08* |
| Malaysia | 50 | 0.74 | 34 | 0.02 | 43 | 0.05 | 46 | 0.36 |
| Mali | *138* | *0.10* | 107 | 0.00 | 90 | 0.00 | *138* | *0.05* |
| Mauritania | 119 | 0.30 | 129 | 0.00 | 117 | 0.00 | 119 | 0.14 |
| Mauritius | 71 | 0.72 | 106 | 0.00 | 30 | 0.17 | 40 | 0.40 |
| Mexico | 30 | 0.82 | **14** | **0.07** | 66 | 0.01 | 37 | 0.40 |
| Mongolia | 99 | 0.58 | 118 | 0.00 | 73 | 0.01 | 100 | 0.26 |
| Montenegro | 47 | 0.75 | 127 | 0.00 | 48 | 0.03 | 53 | 0.35 |
| Morocco | 80 | 0.68 | 54 | 0.01 | 71 | 0.01 | 81 | 0.31 |
| Mozambique | *141* | *0.07* | 109 | 0.00 | 67 | 0.01 | *140* | *0.03* |
| Namibia | 112 | 0.41 | 105 | 0.00 | 40 | 0.06 | 111 | 0.21 |
| Nepal | 97 | 0.59 | 96 | 0.00 | 120 | 0.00 | 99 | 0.26 |
| Netherlands | **12** | **0.93** | 16 | 0.05 | **9** | **0.76** | **11** | **0.78** |
| New Zealand | **12** | **0.93** | 49 | 0.01 | **4** | **0.91** | **5** | **0.83** |
| Nicaragua | 57 | 0.74 | 113 | 0.00 | 106 | 0.00 | 72 | 0.33 |
| Niger | 130 | 0.19 | 122 | 0.00 | 104 | 0.00 | 132 | 0.09 |
| Nigeria | *136* | *0.11* | 37 | 0.02 | 132 | 0.00 | *136* | *0.06* |
| Norway | **9** | **0.94** | 25 | 0.03 | **7** | **0.88** | **6** | **0.83** |
| Pakistan | 106 | 0.50 | 44 | 0.01 | *135* | *0.00* | 107 | 0.23 |
| Panama | 34 | 0.80 | 80 | 0.00 | 54 | 0.03 | 43 | 0.37 |
| Paraguay | 78 | 0.70 | 90 | 0.00 | 108 | 0.00 | 80 | 0.31 |
| Peru | 56 | 0.74 | 47 | 0.01 | 70 | 0.01 | 65 | 0.34 |
| Philippines | 96 | 0.59 | 42 | 0.01 | 98 | 0.00 | 96 | 0.27 |
| Poland | 32 | 0.81 | 21 | 0.03 | 26 | 0.21 | 29 | 0.47 |
| Portugal | 25 | 0.88 | 38 | 0.02 | 22 | 0.28 | 23 | 0.53 |
| Republic of Korea | **15** | **0.93** | **15** | **0.07** | 33 | 0.15 | 25 | 0.51 |
| Republic of Moldova | 92 | 0.60 | 119 | 0.00 | 84 | 0.00 | 95 | 0.27 |
| Romania | 61 | 0.73 | 45 | 0.01 | 45 | 0.04 | 54 | 0.35 |
| Russian Federation | 94 | 0.60 | **11** | **0.11** | 111 | 0.00 | 78 | 0.32 |
| Rwanda | 127 | 0.21 | 121 | 0.00 | 76 | 0.00 | 128 | 0.10 |
| Saint Vincent and the Grenadines | 79 | 0.69 | *146* | *0.00* | 25 | 0.22 | 35 | 0.41 |
| Samoa | 77 | 0.70 | *148* | *0.00* | 42 | 0.05 | 67 | 0.33 |
| Saudi Arabia | 53 | 0.74 | 20 | 0.04 | 89 | 0.00 | 56 | 0.35 |
| Senegal | 117 | 0.32 | 100 | 0.00 | 87 | 0.00 | 117 | 0.15 |
| Serbia | 51 | 0.74 | 74 | 0.00 | 62 | 0.01 | 64 | 0.34 |
| Seychelles | 62 | 0.73 | *141* | *0.00* | 47 | 0.04 | 63 | 0.34 |
| Sierra Leone | *147* | *0.00* | 132 | 0.00 | 112 | 0.00 | *148* | *0.00* |
| Slovakia | 41 | 0.78 | 55 | 0.01 | 28 | 0.19 | 33 | 0.44 |
| Slovenia | 22 | 0.90 | 68 | 0.00 | 24 | 0.25 | 24 | 0.52 |
| Solomon Islands | 101 | 0.56 | *147* | *0.00* | 102 | 0.00 | 102 | 0.25 |
| South Africa | *135* | *0.13* | 29 | 0.03 | 41 | 0.05 | 131 | 0.09 |
| Spain | **5** | **0.96** | **12** | **0.10** | 29 | 0.17 | 20 | 0.55 |
| Sri Lanka | 43 | 0.76 | 66 | 0.00 | 80 | 0.00 | 59 | 0.35 |
| Sudan (Former) | 115 | 0.38 | 58 | 0.00 | *148* | *0.00* | 115 | 0.17 |
| Suriname | 86 | 0.64 | 126 | 0.00 | 64 | 0.01 | 87 | 0.29 |
| Swaziland | *144* | *0.03* | 128 | 0.00 | 92 | 0.00 | *144* | *0.01* |
| Sweden | **7** | **0.95** | 23 | 0.03 | **3** | **0.92** | **3** | **0.85** |
| Switzerland | **2** | **0.97** | 19 | 0.04 | **6** | **0.88** | **4** | **0.85** |
| Syrian Arab Republic | 36 | 0.79 | 60 | 0.00 | 124 | 0.00 | 50 | 0.36 |
| Tajikistan | 102 | 0.56 | 120 | 0.00 | 131 | 0.00 | 103 | 0.25 |
| Thailand | 52 | 0.74 | 30 | 0.02 | 82 | 0.00 | 62 | 0.34 |
| The Former Yugoslav Republic of Macedonia | 44 | 0.76 | 108 | 0.00 | 56 | 0.01 | 57 | 0.35 |
| Timor Leste | 113 | 0.41 | *143* | *0.00* | 125 | 0.00 | 113 | 0.18 |
| Togo | 123 | 0.26 | 131 | 0.00 | 126 | 0.00 | 124 | 0.12 |
| Trinidad and Tobago | 88 | 0.63 | 89 | 0.00 | 53 | 0.03 | 85 | 0.29 |
| Tunisia | 46 | 0.76 | 69 | 0.00 | 68 | 0.01 | 60 | 0.35 |
| Turkey | 58 | 0.74 | 17 | 0.05 | 63 | 0.01 | 49 | 0.36 |
| Turkmenistan | 109 | 0.49 | 86 | 0.00 | *147* | *0.00* | 110 | 0.22 |
| Uganda | 132 | 0.17 | 93 | 0.00 | 97 | 0.00 | 133 | 0.08 |
| Ukraine | 87 | 0.64 | 50 | 0.01 | 99 | 0.00 | 89 | 0.29 |
| United Kingdom | 16 | 0.92 | **6** | **0.16** | **15** | **0.49** | 17 | 0.70 |
| United Republic of Tanzania | 121 | 0.28 | 85 | 0.00 | 77 | 0.00 | 122 | 0.13 |
| United States of America | 27 | 0.87 | **1** | **1.00** | 19 | 0.36 | **1** | **1.00** |
| Uruguay | 33 | 0.81 | 72 | 0.00 | 27 | 0.20 | 30 | 0.45 |
| Uzbekistan | 100 | 0.58 | 71 | 0.00 | *144* | *0.00* | 101 | 0.26 |
| Vanuatu | 83 | 0.66 | *145* | *0.00* | 49 | 0.03 | 83 | 0.31 |
| Venezuela (Bolivarian Republic of) | 49 | 0.75 | 26 | 0.03 | *143* | *0.00* | 58 | 0.35 |
| Yemen | 108 | 0.49 | 78 | 0.00 | *136* | *0.00* | 108 | 0.22 |
| Zambia | *143* | *0.03* | 95 | 0.00 | 83 | 0.00 | *143* | *0.01* |

**Table S5.** Vulnerability, sensitivity, exposure and adaptive capacity rank and scores for all nations included in the vulnerability analysis of livestock-based food production, scaled from zero to one. Bold indicates upper tenth percentile, italics indicates lower tenth percentiles.

|  | **Vulnerability** | | **Sensitivity** | | **Exposure** | | **Adaptive Capacity** | | **Vulnerability (to climate change only)** | | **Vulnerability (to population growth only)** | |
| --- | --- | --- | --- | --- | --- | --- | --- | --- | --- | --- | --- | --- |
| **Nation** | **Rank** | **Score** | **Rank** | **Score** | **Rank** | **Score** | **Rank** | **Score** | **Rank** | **Score** | **Rank** | **Score** |
| Albania | 19 | 0.70 | **12** | **0.63** | **4** | **0.47** | 42 | 0.37 | **5** | **0.88** | 70 | 0.49 |
| Algeria | 78 | 0.41 | 87 | 0.34 | 77 | 0.20 | 76 | 0.32 | 83 | 0.39 | 71 | 0.48 |
| Angola | 18 | 0.71 | 53 | 0.43 | 21 | 0.36 | *139* | *0.04* | 29 | 0.62 | 14 | 0.84 |
| Argentina | 57 | 0.50 | 16 | 0.62 | 101 | 0.13 | 45 | 0.37 | 45 | 0.53 | 53 | 0.58 |
| Armenia | 77 | 0.41 | 26 | 0.52 | 129 | 0.02 | 68 | 0.33 | 52 | 0.51 | 82 | 0.44 |
| Australia | 123 | 0.19 | 58 | 0.41 | 86 | 0.17 | **14** | **0.76** | 130 | 0.19 | 123 | 0.22 |
| Austria | *146* | *0.05* | 106 | 0.29 | 118 | 0.05 | **12** | **0.77** | *145* | *0.09* | *146* | *0.05* |
| Azerbaijan | 55 | 0.51 | 30 | 0.51 | 85 | 0.17 | 88 | 0.29 | 46 | 0.53 | 55 | 0.57 |
| Bangladesh | 67 | 0.44 | 88 | 0.33 | 75 | 0.21 | 97 | 0.27 | 73 | 0.43 | 64 | 0.52 |
| Belarus | 108 | 0.29 | 103 | 0.29 | *141* | *0.00* | 90 | 0.29 | 85 | 0.38 | 107 | 0.33 |
| Belgium | *138* | *0.11* | 110 | 0.28 | 122 | 0.05 | 18 | 0.65 | *134* | *0.16* | *138* | *0.12* |
| Belize | 73 | 0.43 | 129 | 0.20 | **12** | **0.40** | 47 | 0.36 | 101 | 0.33 | 68 | 0.49 |
| Benin | 45 | 0.56 | *137* | *0.18* | **13** | **0.40** | 127 | 0.10 | 64 | 0.46 | 40 | 0.65 |
| Bolivia (Plurinational State of) | 26 | 0.66 | **15** | **0.63** | 52 | 0.26 | 106 | 0.24 | 21 | 0.64 | 23 | 0.75 |
| Bosnia and Herzegovina | 95 | 0.34 | 63 | 0.40 | 126 | 0.03 | 52 | 0.35 | 72 | 0.43 | 100 | 0.36 |
| Botswana | 16 | 0.72 | **9** | **0.64** | 55 | 0.25 | 121 | 0.13 | **12** | **0.71** | 17 | 0.82 |
| Brazil | 80 | 0.40 | 44 | 0.44 | 90 | 0.16 | 36 | 0.41 | 75 | 0.41 | 78 | 0.46 |
| Bulgaria | 106 | 0.30 | 73 | 0.36 | *144* | *0.00* | 55 | 0.35 | 82 | 0.39 | 106 | 0.33 |
| Burkina Faso | 24 | 0.67 | 71 | 0.37 | **9** | **0.41** | 129 | 0.10 | 32 | 0.61 | 24 | 0.75 |
| Burundi | **2** | **0.93** | **5** | **0.75** | **6** | **0.42** | *141* | *0.03* | **2** | **0.95** | **3** | **0.96** |
| Côte d'Ivoire | 47 | 0.54 | 111 | 0.28 | 48 | 0.26 | 130 | 0.09 | 55 | 0.49 | 41 | 0.65 |
| Cambodia | 54 | 0.51 | 93 | 0.31 | 49 | 0.26 | 112 | 0.19 | 60 | 0.48 | 54 | 0.58 |
| Cameroon | 35 | 0.60 | 91 | 0.33 | 45 | 0.28 | *137* | *0.05* | 40 | 0.55 | 30 | 0.71 |
| Canada | *144* | *0.06* | 118 | 0.26 | 104 | 0.12 | **10** | **0.79** | *146* | *0.06* | *143* | *0.08* |
| Cape Verde | 82 | 0.39 | 83 | 0.34 | 74 | 0.21 | 44 | 0.37 | 93 | 0.36 | 77 | 0.47 |
| Central African Republic | **13** | **0.76** | 17 | 0.61 | 63 | 0.23 | *147* | *0.00* | **9** | **0.75** | **8** | **0.89** |
| Chad | **8** | **0.79** | 27 | 0.51 | 10 | 0.41 | *142* | *0.02* | **13** | **0.71** | **5** | **0.91** |
| Chile | 114 | 0.25 | 85 | 0.34 | 91 | 0.16 | 19 | 0.57 | 124 | 0.25 | 113 | 0.29 |
| China | 102 | 0.31 | 114 | 0.26 | 44 | 0.29 | 26 | 0.51 | 90 | 0.37 | 121 | 0.24 |
| Colombia | 27 | 0.65 | **4** | **0.75** | 64 | 0.23 | 66 | 0.34 | 20 | 0.66 | 25 | 0.74 |
| Congo | 65 | 0.46 | *144* | *0.11* | 36 | 0.31 | 123 | 0.12 | 86 | 0.38 | 61 | 0.55 |
| Croatia | 116 | 0.23 | 102 | 0.29 | *145* | *0.00* | 38 | 0.40 | 107 | 0.32 | 118 | 0.26 |
| Cyprus | 92 | 0.35 | 47 | 0.43 | 72 | 0.21 | 21 | 0.54 | 109 | 0.32 | 86 | 0.42 |
| Czech Republic | 122 | 0.19 | 97 | 0.31 | 130 | 0.01 | 28 | 0.50 | 117 | 0.27 | 124 | 0.21 |
| Denmark | *145* | *0.05* | 77 | 0.35 | 123 | 0.05 | **7** | **0.83** | *144* | *0.10* | *145* | *0.06* |
| Dominican Republic | 62 | 0.47 | 51 | 0.43 | 70 | 0.21 | 74 | 0.33 | 66 | 0.45 | 59 | 0.55 |
| Ecuador | 46 | 0.55 | 20 | 0.58 | 62 | 0.23 | 51 | 0.35 | 41 | 0.55 | 47 | 0.62 |
| Egypt | 86 | 0.36 | 121 | 0.25 | 73 | 0.21 | 73 | 0.33 | 98 | 0.34 | 83 | 0.44 |
| El Salvador | 76 | 0.42 | 55 | 0.42 | 106 | 0.11 | 82 | 0.31 | 63 | 0.47 | 80 | 0.46 |
| Eritrea | **3** | **0.91** | **3** | **0.89** | **15** | **0.39** | 116 | 0.17 | **4** | **0.88** | **2** | **1.00** |
| Estonia | 110 | 0.29 | 33 | 0.49 | *146* | *0.00* | 27 | 0.50 | 87 | 0.38 | 109 | 0.32 |
| Ethiopia | **14** | **0.75** | 18 | 0.61 | 23 | 0.36 | 118 | 0.14 | 16 | 0.69 | **11** | **0.86** |
| Fiji | 83 | 0.39 | 112 | 0.27 | 83 | 0.18 | 94 | 0.27 | 68 | 0.44 | 93 | 0.39 |
| Finland | *141* | *0.08* | 48 | 0.43 | 121 | 0.05 | **2** | **0.86** | *140* | *0.13* | *142* | *0.09* |
| France | 131 | 0.16 | 60 | 0.41 | 113 | 0.06 | 16 | 0.70 | 129 | 0.20 | 133 | 0.18 |
| Gambia | 58 | 0.50 | *140* | *0.17* | 27 | 0.34 | 120 | 0.14 | 77 | 0.41 | 48 | 0.61 |
| Georgia | 60 | 0.48 | **13** | **0.63** | 125 | 0.03 | 69 | 0.33 | 34 | 0.58 | 65 | 0.51 |
| Germany | *143* | *0.06* | 84 | 0.34 | 131 | 0.01 | **13** | **0.77** | *141* | *0.12* | *144* | *0.06* |
| Ghana | 79 | 0.40 | *146* | *0.10* | 32 | 0.32 | 109 | 0.22 | 100 | 0.33 | 74 | 0.47 |
| Greece | 113 | 0.27 | 75 | 0.36 | 112 | 0.06 | 31 | 0.45 | 105 | 0.32 | 112 | 0.31 |
| Guatemala | 38 | 0.58 | 46 | 0.44 | 18 | 0.37 | 84 | 0.30 | 43 | 0.54 | 45 | 0.63 |
| Guinea | 39 | 0.58 | 108 | 0.28 | 34 | 0.31 | *135* | *0.08* | 54 | 0.50 | 34 | 0.69 |
| Guinea-Bissau | 41 | 0.57 | 116 | 0.26 | 59 | 0.24 | *146* | *0.00* | 42 | 0.54 | 37 | 0.66 |
| Guyana | 59 | 0.48 | 101 | 0.30 | 33 | 0.32 | 91 | 0.28 | 31 | 0.61 | 101 | 0.36 |
| Haiti | 31 | 0.62 | 24 | 0.55 | 68 | 0.22 | 114 | 0.18 | 28 | 0.62 | 32 | 0.71 |
| Honduras | 50 | 0.52 | 68 | 0.38 | 24 | 0.35 | 77 | 0.32 | 56 | 0.49 | 58 | 0.56 |
| Hungary | 129 | 0.17 | 130 | 0.20 | *139* | *0.00* | 34 | 0.41 | 121 | 0.26 | 131 | 0.19 |
| India | 40 | 0.58 | 32 | 0.49 | 40 | 0.29 | 92 | 0.28 | 36 | 0.57 | 46 | 0.62 |
| Indonesia | 101 | 0.32 | *136* | *0.18* | 93 | 0.15 | 86 | 0.29 | 106 | 0.32 | 96 | 0.37 |
| Iran (Islamic Republic of) | 68 | 0.43 | 86 | 0.34 | 57 | 0.25 | 70 | 0.33 | 71 | 0.44 | 79 | 0.46 |
| Ireland | 126 | 0.18 | 67 | 0.38 | 97 | 0.14 | **15** | **0.71** | 131 | 0.18 | 122 | 0.22 |
| Israel | 91 | 0.35 | 113 | 0.26 | 42 | 0.29 | 32 | 0.45 | 116 | 0.27 | 84 | 0.43 |
| Italy | 130 | 0.17 | 104 | 0.29 | 124 | 0.03 | 22 | 0.54 | 125 | 0.23 | 127 | 0.19 |
| Jamaica | 89 | 0.36 | 89 | 0.33 | 103 | 0.12 | 75 | 0.33 | 78 | 0.40 | 91 | 0.40 |
| Japan | *148* | *0.00* | 119 | 0.25 | 128 | 0.02 | **9** | **0.80** | *147* | *0.06* | *148* | *0.00* |
| Jordan | 64 | 0.46 | 120 | 0.25 | 16 | 0.37 | 71 | 0.33 | 95 | 0.34 | 57 | 0.56 |
| Kazakhstan | 71 | 0.43 | 29 | 0.51 | *137* | *0.00* | 98 | 0.27 | 48 | 0.53 | 72 | 0.48 |
| Kenya | **1** | **1.00** | **7** | **0.70** | **1** | **0.67** | 125 | 0.12 | **1** | **1.00** | **4** | **0.94** |
| Kuwait | 109 | 0.29 | 131 | 0.20 | 87 | 0.16 | 48 | 0.36 | 115 | 0.28 | 104 | 0.35 |
| Kyrgyzstan | 43 | 0.56 | **10** | **0.64** | 102 | 0.12 | 93 | 0.27 | 33 | 0.60 | 42 | 0.65 |
| Lao People's Democratic Republic | 52 | 0.52 | 65 | 0.39 | 53 | 0.26 | 104 | 0.25 | 58 | 0.49 | 52 | 0.59 |
| Latvia | 112 | 0.28 | 72 | 0.37 | *146* | *0.00* | 41 | 0.39 | 91 | 0.37 | 111 | 0.31 |
| Lebanon | 74 | 0.43 | 80 | 0.35 | 71 | 0.21 | 79 | 0.31 | 79 | 0.40 | 66 | 0.50 |
| Lesotho | 33 | 0.61 | 69 | 0.38 | 78 | 0.20 | *145* | *0.00* | 23 | 0.63 | 35 | 0.68 |
| Liberia | 32 | 0.61 | 105 | 0.29 | **14** | **0.39** | 126 | 0.11 | 53 | 0.51 | 29 | 0.72 |
| Libya | 99 | 0.33 | 133 | 0.19 | 69 | 0.22 | 61 | 0.34 | 114 | 0.29 | 92 | 0.40 |
| Lithuania | 115 | 0.24 | 100 | 0.30 | *146* | *0.00* | 39 | 0.40 | 103 | 0.33 | 116 | 0.26 |
| Luxembourg | 133 | 0.15 | 61 | 0.40 | 88 | 0.16 | **8** | **0.81** | *139* | *0.14* | 130 | 0.19 |
| Madagascar | 25 | 0.67 | 25 | 0.55 | 22 | 0.36 | 105 | 0.24 | 35 | 0.58 | 22 | 0.79 |
| Malawi | 28 | 0.65 | 96 | 0.31 | **8** | **0.41** | *134* | *0.08* | 24 | 0.63 | 36 | 0.68 |
| Malaysia | 117 | 0.23 | *148* | *0.00* | 54 | 0.25 | 46 | 0.36 | 133 | 0.17 | 114 | 0.29 |
| Mali | 20 | 0.70 | 62 | 0.40 | 19 | 0.37 | *138* | *0.05* | 27 | 0.62 | 20 | 0.81 |
| Mauritania | **12** | **0.76** | 36 | 0.48 | **3** | **0.49** | 119 | 0.14 | **11** | **0.71** | 21 | 0.79 |
| Mauritius | 118 | 0.23 | *139* | *0.17* | 105 | 0.11 | 40 | 0.40 | 123 | 0.25 | 115 | 0.27 |
| Mexico | 94 | 0.34 | 95 | 0.31 | 82 | 0.18 | 37 | 0.40 | 97 | 0.34 | 90 | 0.40 |
| Mongolia | **6** | **0.81** | **1** | **1.00** | 79 | 0.20 | 100 | 0.26 | **6** | **0.87** | **7** | **0.89** |
| Montenegro | 87 | 0.36 | 42 | 0.45 | 127 | 0.02 | 53 | 0.35 | 69 | 0.44 | 87 | 0.41 |
| Morocco | 93 | 0.35 | 117 | 0.26 | 95 | 0.15 | 81 | 0.31 | 94 | 0.35 | 88 | 0.41 |
| Mozambique | 21 | 0.69 | 54 | 0.42 | 30 | 0.32 | *140* | *0.03* | 26 | 0.62 | 19 | 0.81 |
| Namibia | **15** | **0.74** | **8** | **0.66** | 25 | 0.35 | 111 | 0.21 | **14** | **0.71** | 16 | 0.82 |
| Nepal | 44 | 0.56 | 50 | 0.43 | 37 | 0.31 | 99 | 0.26 | 50 | 0.52 | 43 | 0.64 |
| Netherlands | *135* | *0.13* | 49 | 0.43 | 115 | 0.06 | **11** | **0.78** | 132 | 0.17 | *135* | *0.15* |
| New Zealand | *134* | *0.15* | 45 | 0.44 | 98 | 0.14 | **5** | **0.83** | *137* | *0.15* | 132 | 0.19 |
| Nicaragua | 53 | 0.51 | 38 | 0.47 | 51 | 0.26 | 72 | 0.33 | 51 | 0.51 | 56 | 0.56 |
| Niger | **9** | **0.79** | 43 | 0.45 | **2** | **0.52** | 132 | 0.09 | **15** | **0.70** | 13 | 0.86 |
| Nigeria | 56 | 0.51 | *142* | *0.16* | 46 | 0.28 | *136* | *0.06* | 67 | 0.45 | 50 | 0.60 |
| Norway | *142* | *0.07* | 74 | 0.36 | 109 | 0.08 | **6** | **0.83** | *143* | *0.10* | *141* | *0.09* |
| Pakistan | 17 | 0.71 | **6** | **0.71** | 47 | 0.27 | 107 | 0.23 | 19 | 0.68 | **15** | **0.82** |
| Panama | 61 | 0.47 | 40 | 0.46 | 65 | 0.23 | 43 | 0.37 | 70 | 0.44 | 60 | 0.55 |
| Paraguay | 51 | 0.52 | 41 | 0.46 | 50 | 0.26 | 80 | 0.31 | 59 | 0.49 | 49 | 0.61 |
| Peru | 63 | 0.46 | 76 | 0.36 | 43 | 0.29 | 65 | 0.34 | 61 | 0.48 | 75 | 0.47 |
| Philippines | 85 | 0.38 | *138* | *0.18* | 60 | 0.24 | 96 | 0.27 | 102 | 0.33 | 81 | 0.45 |
| Poland | 124 | 0.19 | 107 | 0.28 | *142* | *0.00* | 29 | 0.47 | 118 | 0.27 | 126 | 0.20 |
| Portugal | 132 | 0.15 | 124 | 0.24 | 120 | 0.05 | 23 | 0.53 | 127 | 0.21 | *134* | *0.18* |
| Republic of Korea | *137* | *0.11* | *145* | *0.10* | 108 | 0.09 | 25 | 0.51 | *138* | *0.14* | *137* | *0.13* |
| Republic of Moldova | 70 | 0.43 | 28 | 0.51 | *135* | *0.01* | 95 | 0.27 | 47 | 0.53 | 73 | 0.48 |
| Romania | 100 | 0.32 | 64 | 0.39 | *138* | *0.00* | 54 | 0.35 | 76 | 0.41 | 102 | 0.35 |
| Russian Federation | 90 | 0.35 | 56 | 0.42 | *136* | *0.01* | 78 | 0.32 | 65 | 0.45 | 94 | 0.39 |
| Rwanda | 22 | 0.69 | 39 | 0.46 | 26 | 0.34 | 128 | 0.10 | 17 | 0.68 | 27 | 0.73 |
| Saint Vincent and the Grenadines | 128 | 0.17 | *134* | *0.19* | 133 | 0.01 | 35 | 0.41 | 122 | 0.25 | 128 | 0.19 |
| Samoa | 121 | 0.21 | *143* | *0.13* | 111 | 0.07 | 67 | 0.33 | 120 | 0.26 | 120 | 0.25 |
| Saudi Arabia | 81 | 0.40 | 125 | 0.23 | 35 | 0.31 | 56 | 0.35 | 111 | 0.31 | 69 | 0.49 |
| Senegal | 34 | 0.61 | 70 | 0.38 | 28 | 0.33 | 117 | 0.15 | 44 | 0.54 | 31 | 0.71 |
| Serbia | 97 | 0.33 | 59 | 0.41 | *143* | *0.00* | 64 | 0.34 | 74 | 0.42 | 97 | 0.37 |
| Seychelles | 120 | 0.22 | *147* | *0.09* | 100 | 0.13 | 63 | 0.34 | 126 | 0.23 | 117 | 0.26 |
| Sierra Leone | 29 | 0.63 | 66 | 0.38 | 67 | 0.22 | *148* | *0.00* | 30 | 0.61 | 26 | 0.74 |
| Slovakia | 119 | 0.23 | 99 | 0.31 | 132 | 0.01 | 33 | 0.44 | 113 | 0.31 | 119 | 0.25 |
| Slovenia | 125 | 0.18 | 94 | 0.31 | 133 | 0.01 | 24 | 0.52 | 119 | 0.26 | 125 | 0.21 |
| Solomon Islands | 72 | 0.43 | *141* | *0.17* | 29 | 0.33 | 102 | 0.25 | 99 | 0.34 | 63 | 0.52 |
| South Africa | 49 | 0.53 | 109 | 0.28 | 58 | 0.24 | 131 | 0.09 | 49 | 0.52 | 51 | 0.60 |
| Spain | 127 | 0.18 | 128 | 0.21 | 96 | 0.14 | 20 | 0.55 | 128 | 0.21 | 129 | 0.19 |
| Sri Lanka | 69 | 0.43 | 81 | 0.35 | 56 | 0.25 | 59 | 0.35 | 57 | 0.49 | 89 | 0.41 |
| Sudan (Former) | **4** | **0.87** | **2** | **0.89** | 31 | 0.32 | 115 | 0.17 | **7** | **0.83** | **1** | **1.00** |
| Suriname | 88 | 0.36 | 115 | 0.26 | 92 | 0.15 | 87 | 0.29 | 92 | 0.37 | 85 | 0.42 |
| Swaziland | **5** | **0.85** | **11** | **0.64** | 17 | 0.37 | *144* | *0.01* | **3** | **0.90** | **10** | **0.86** |
| Sweden | *136* | *0.11* | 37 | 0.47 | 116 | 0.06 | **3** | **0.85** | *136* | *0.16* | *136* | *0.13* |
| Switzerland | *140* | *0.10* | 57 | 0.42 | 107 | 0.09 | **4** | **0.85** | *142* | *0.12* | *140* | *0.12* |
| Syrian Arab Republic | 66 | 0.44 | 92 | 0.32 | 38 | 0.31 | 50 | 0.36 | 89 | 0.38 | 62 | 0.53 |
| Tajikistan | 36 | 0.60 | 19 | 0.60 | 76 | 0.21 | 103 | 0.25 | 22 | 0.63 | 38 | 0.66 |
| Thailand | 107 | 0.30 | 132 | 0.19 | 89 | 0.16 | 62 | 0.34 | 104 | 0.33 | 110 | 0.32 |
| The Former Yugoslav Republic of Macedonia | 96 | 0.33 | 82 | 0.34 | 110 | 0.07 | 57 | 0.35 | 80 | 0.40 | 99 | 0.36 |
| Timor Leste | 37 | 0.60 | 34 | 0.49 | 61 | 0.24 | 113 | 0.18 | 37 | 0.57 | 33 | 0.70 |
| Togo | 48 | 0.54 | 122 | 0.25 | 39 | 0.30 | 124 | 0.12 | 62 | 0.47 | 44 | 0.63 |
| Trinidad and Tobago | 111 | 0.28 | 127 | 0.22 | 114 | 0.06 | 85 | 0.29 | 96 | 0.34 | 108 | 0.32 |
| Tunisia | 104 | 0.31 | 126 | 0.22 | 94 | 0.15 | 60 | 0.35 | 110 | 0.31 | 98 | 0.36 |
| Turkey | 98 | 0.33 | 123 | 0.24 | 84 | 0.18 | 49 | 0.36 | 108 | 0.32 | 95 | 0.38 |
| Turkmenistan | 30 | 0.62 | **14** | **0.63** | 81 | 0.18 | 110 | 0.22 | 25 | 0.63 | 28 | 0.73 |
| Uganda | **11** | **0.77** | 35 | 0.49 | **5** | **0.44** | 133 | 0.08 | 18 | 0.68 | **9** | **0.87** |
| Ukraine | 105 | 0.30 | 98 | 0.31 | *140* | *0.00* | 89 | 0.29 | 81 | 0.39 | 105 | 0.34 |
| United Kingdom | *139* | *0.11* | 90 | 0.33 | 119 | 0.05 | 17 | 0.70 | *135* | *0.16* | *139* | *0.12* |
| United Republic of Tanzania | **10** | **0.78** | 21 | 0.58 | **7** | **0.42** | 122 | 0.13 | **10** | **0.72** | **12** | **0.86** |
| United States of America | *147* | *0.00* | 78 | 0.35 | 99 | 0.13 | **1** | **1.00** | *148* | *0.00* | *147* | *0.01* |
| Uruguay | 103 | 0.31 | 52 | 0.43 | 117 | 0.06 | 30 | 0.45 | 88 | 0.38 | 103 | 0.35 |
| Uzbekistan | 42 | 0.56 | 23 | 0.56 | 80 | 0.18 | 101 | 0.26 | 38 | 0.56 | 39 | 0.66 |
| Vanuatu | 84 | 0.38 | *135* | *0.18* | 41 | 0.29 | 83 | 0.31 | 112 | 0.31 | 76 | 0.47 |
| Venezuela (Bolivarian Republic of) | 75 | 0.42 | 79 | 0.35 | 66 | 0.22 | 58 | 0.35 | 84 | 0.39 | 67 | 0.50 |
| Yemen | 23 | 0.68 | 31 | 0.50 | **11** | **0.40** | 108 | 0.22 | 39 | 0.55 | 18 | 0.81 |
| Zambia | **7** | **0.81** | 22 | 0.57 | 20 | 0.37 | *143* | *0.01* | **8** | **0.77** | **6** | **0.90** |
